# Structures of calmodulin–melittin complexes show multiple binding modes lacking classical anchoring interactions

**DOI:** 10.1016/j.jbc.2023.104596

**Published:** 2023-03-09

**Authors:** Zsolt Dürvanger, Tünde Juhász, Károly Liliom, Veronika Harmat

**Affiliations:** 1 Laboratory of Structural Chemistry and Biology, Institute of Chemistry, ELTE Eötvös Loránd University, Budapest, Hungary; 2 Institute of Materials and Environmental Chemistry, Research Centre for Natural Sciences, Budapest, Hungary; 3 Department of Biophysics and Radiation Biology, Faculty of Medicine, Semmelweis University, Budapest, Hungary; 4 ELKH-ELTE Protein Modelling Research Group, Eötvös Loránd Research Network, Budapest, Hungary

**Keywords:** calmodulin (CaM), protein–protein interaction, protein complex, crystal structure, molecular dynamics, multiple binding modes, linear motif-binding hub protein, polymorphic protein–protein complex, fuzzy complex

## Abstract

Calmodulin (CaM) is a Ca^2+^ sensor protein found in all eukaryotic cells that regulates a large number of target proteins in a Ca^2+^ concentration-dependent manner. As a transient-type hub protein, it recognizes linear motifs of its targets, though for the Ca^2+^-dependent binding, no consensus sequence was identified. Its complex with melittin, a major component of bee venom, is often used as a model system of protein–protein complexes. Yet, the structural aspects of the binding are not well understood, as only diverse, low-resolution data are available concerning the association. We present the crystal structure of melittin in complex with Ca^2+^-saturated CaMs from two, evolutionarily distant species, *Homo sapiens* and *Plasmodium falciparum*, representing three binding modes of the peptide. Results—augmented by molecular dynamics simulations—indicate that multiple binding modes can exist for CaM–melittin complexes, as an intrinsic characteristic of the binding. While the helical structure of melittin remains, swapping of its salt bridges and partial unfolding of its C-terminal segment can occur. In contrast to the classical way of target recognition by CaM, we found that different sets of residues can anchor at the hydrophobic pockets of CaM, which were considered as main recognition sites. Finally, the nanomolar binding affinity of the CaM–melittin complex is created by an ensemble of arrangements of similar stability—tight binding is achieved not by optimized specific interactions but by simultaneously satisfying less optimal interaction patterns in co-existing different conformers.

Hub proteins are crucial for functioning of all living organisms. As they interact with many partner proteins, defects in these interactions such as losing or gaining (abnormal) binding affinity or alteration of binding-caused conformational changes can be lethal (1). A common characteristic of hub proteins is flexibility that plays a key role in their promiscuous nature—this can manifest in various forms, as flexible loops of a folded protein adopting to different binding partners, a flexible linker between their folded domains ensuring shape adaptation to different binding partners, or intrinsical disorder, in some cases extending to the whole protein, creating multiple binding sites. These flexible parts often contain the partner recognition site which is stabilized within the complex (2, 3). There are two main categories of hubs differentiated based on their role in protein networks (that also result in some characteristic structural features); some bind multiple partners in a stationary fashion to their multiple binding sites (obligate or party hub proteins), others bind several partners in a transient fashion with one binding site (transient or date hub proteins) (4). The latter group is also referred to as linear motif binding hub proteins. The name refers to the curious structural property of binding: the binding partners compete for the same binding site of the hub protein, which recognizes short linear sequence motifs on the partners (5), *e.g.*, short, functional regions that carry a particular sequence. Binding of the sequence motifs with not strictly limited sequence patterns by the binding site of the highly conserved hub protein ensures the fine balance between specificity (binding only its targets) and promiscuity of the hub protein. To understand these dual aspects of hub protein target recognition, detailed structural characterization is required, which can be demanding due to the flexibility and variability of these systems.

Calmodulin (CaM), which binds to and alters the action of over 200 proteins, recognizing their segments of highly diverse sequences, is an often-cited example of transient type of hub proteins (4). It is a small calcium-binding protein that plays a key role in the signal transduction pathways of all eukaryotic cells (6). The amino acid sequence of CaM is highly conserved, showing 100% identity in all mammals. The protein consists of two homologous domains (also called the N-terminal and C-terminal lobes), containing two Ca^2+^-binding EF-hand structures each, connected by a flexible central linker (7, 8). The two lobes of the protein undergo large conformational changes upon calcium binding, as a consequence of which its target binding hydrophobic residues become solvent accessible (open conformation of the lobes). This creates the basis of calcium-dependent interactions with its target proteins, allowing CaM to function as a calcium-dependent molecular switch (6).

While apo-CaM (*i.e.*, Ca^2+^-free CaM) is also involved in forming complexes by specifically binding IQ motifs (a generalized form is: [I,L,V]QXXXRXXXX[R,K]) (9, 10), the calcium-dependent binding of partners carry a more diverse set of binding sequences with no obviously conserved elements. Although interaction can also depend on the number of loaded Ca^2+^ ions (11), here we focus on interactions of CaM saturated with Ca^2+^-ions, as most structural studies describe these complexes. The interaction of CaM with its targets is characterized in many cases by CaM binding peptides consisting of the binding segments of its targets. Although a common sequence pattern of CaM-binding cannot be established, a common structural property could be observed: target peptides are able to form amphiphilic α-helices upon binding to CaM (12). Furthermore, the majority of CaM binding peptides contain bulky, hydrophobic anchoring residues (*e.g.*, Phe, Trp, Leu or Ile) which fit into the hydrophobic pockets of the two lobes of CaM. The protein usually forms 1:1 complexes with its targets, wrapping around the coordinated peptides, assisted by the flexibility of its central linker. It is notable that in addition to these conventional CaM–peptide complexes, some examples of complexes with 2:2 (13) and 2:1 (14) stoichiometry are also known.

Conventional Ca^2+^/CaM–peptide complexes can be classified based on the number of residues separating the two anchoring residues (15). The most common CaM binding motifs are the 1-10/1-14 motifs, in which there are 8 and 12 residues separating the two anchors, respectively. Due to the high flexibility of CaM and the high variety of CaM binding sequences, several other, unusual binding motifs (*e.g.*, 1–11, 1–17, and 1–18) could also be found (reviewed in (9, 16)). Ca^2+^/CaM–peptide complexes can be also classified based on the binding orientation of the peptide. In the majority of CaM–peptide complexes, the peptide binds in antiparallel orientation, that is the C-terminal anchoring residue of the peptide binds to the N-terminal hydrophobic pocket of CaM, and the N-terminal anchoring residue binds to the C-terminal pocket. Some peptides, however, bind in a reversed, parallel orientation (17, 18, 19) (PDB codes: 1IQ5, 2F3Y, 2BE6). In target peptides, a cluster of basic residues either before the N-terminal or after the C-terminal anchoring residue may dictate the orientation of the peptide (20, 21, 22), though some exceptions can be found to this rule too (16). (As examples, topologies of complexes with 1-11/1-18 binding motifs bound in antiparallel orientations are presented in Fig. 1).

**Figure 1:**
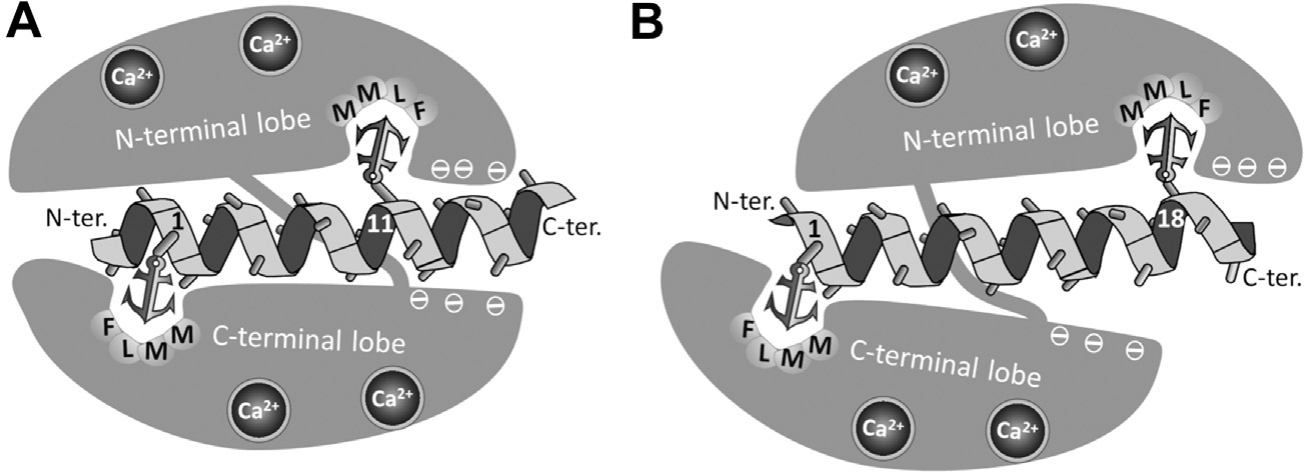
**Topology of conventional CaM - peptide complexes (schematic representation).** In the two examples shown, the helical peptides bound to CaM in antiparallel orientations anchoring by a 1-11 (*A*) and a 1-18 (*B*) motif, respectively.

Melittin, a major component of bee venom, is a 26-residue lytic peptide, with the amino acid sequence GIGAVLKVLTTGLPALISWIKRKRQQ. Under physiological conditions, it is unstructured (23), but it can adopt various helical conformations in different solvents, in trifluoroethanol-containing solution, at higher peptide concentration, or upon associating to cell membranes and proteins (24, 25). In its crystal structure, it forms tetramers of helices stabilized by four sulphate ions (26). The tetrameric associate is also stable in solutions with high ionic strength and higher peptide concentration (27). One of the several proteins that induce helical conversion in melittin upon complex formation is CaM, which binds melittin in the presence of Ca^2+^ with a *K*_d_ in the low nanomolar range (∼3–60 nM) (28, 29, 30) and forms a complex with 1:1 stoichiometry. The sequence of melittin does not contain an IQ motif, and its interaction with CaM is highly Ca^2+^ dependent. While apo-CaM was also reported to form a low affinity complex with melittin, Ca^2+^ is necessary for the formation of a high affinity complex with compact structure (31). Based on sequence comparison of CaM target sequences and fluorescent spectroscopic results, Trp19 was suggested as one of the anchoring residues (21, 32); however, ambiguity regarding the anchoring residues remained in further experimental studies of the complex (21, 33).

Several biological effects of melittin were reported, such as antimicrobial, antibacterial (34), antiviral (35), anti-inflammatory (36), and anticancer properties (37), some of which are attributed to its CaM antagonist properties (38, 39, 40, 41). Due to its cytotoxicity, the potential use of melittin and its conjugates in cancer therapy is also investigated (42, 43). The CaM–melittin complex is often used as a model system in the development of techniques suitable for structural investigations of protein–protein complexes (44, 45, 46). Mass spectrometry combined with chemical cross-linking and limited proteolysis (21, 33, 47), with oxidation by hydroxyl radicals (48), or most recently LITPOMS (49), was also applied to study the structure of this complex. Melittin has been also widely used in characterizing CaM function, target binding and antagonist binding (50, 51, 52, 53, 54), as a model peptide, which is, however, greatly hindered by the fact that no atomic resolution structures of the complex has been determined thus far. The single structure that might provide some insight into the mode of the association (in fact, the single structure describing a protein–melittin complex in the PDB) is that of its complex formed with another member of the EF-hand superfamily of calcium-binding proteins, the *Chlamydomonas reinhardtii* centrin–melittin complex.

The structural characteristics of the CaM–melittin complex were studied using different techniques leading to contradicting results: SAXS study of the complex revealed that the complex adopts a globular structure in contrast to the dumbbell shape of Ca^2+^/CaM (31). H^1^ NMR spectroscopic data showed that both lobes of the protein participate in the binding of melittin (55). A fluorescent spectroscopic study showed that the microenvironment of Trp19 is significantly changed upon complex formation and that Trp19 is located near the C-terminal lobe of CaM (32). This suggested that melittin binds in parallel orientation to CaM and that the C-terminal anchoring residue might be Trp19. High-pressure dissociation study indicated that the spatial fitting is not the tightest possible, suggesting the steric fit is non-ideal (54). Mapping solvent accessible area with photochemically active reagent labeling demonstrated that the N-terminal lobe of CaM and linker region of CaM are more involved in the interaction than the C-terminal lobe (46). Our stopped-flow kinetic study indicated the high-affinity binding of melittin to the N-terminal lobe first, followed by a conformational re-arrangement in the complex, resulting in a closed conformation in which melittin is interacting with both lobes of CaM (30). MS analysis of digestion products after using different chemical cross-linkers concluded that melittin can bind in both parallel and antiparallel orientations to CaM (33) and the preferred orientation is parallel (47). Using OH^.^radical probe, it was showed that complexation shields the N-terminal domain of CaM more effectively than the C-terminal domain, while for melittin, the C-terminal part was more protected, suggesting the stronger involvement of these regions in interactions (48).

The low resolution of the structural information that can be derived from these studies, does not allow full understanding of the binding mode of melittin on CaM or building a consensus model of the binding—which is, in itself, rather surprising, considering the strength of the association. The possibility of multiple binding modes for a particular peptide orientation should be also considered—as it is supported by some of the previous results (33). Furthermore, our previous comparative microcalorimetric studies on human CaM (hCaM) and CaM from the malaria parasite *Plasmodium falciparum* (pfCaM, a potential antimalarial target (56, 57), sharing a 89% identity with hCaM) revealed that even minor variations of the CaM sequence can result in notable differences in its interaction with melittin: while the interaction of melittin with both CaMs could be characterized by positive binding enthalpies, Δ*H* values obtained for pfCaM were somewhat smaller than values for hCaM. Moreover, the binding curves suggested a more complex binding event for hCaM, which could not be observed with pfCaM (29).

Here, we present the crystal structures of hCaM and pfCaM complexed with melittin. To characterize flexibility of the complexes and possibility of shifts between binding modes, molecular dynamics simulations were also carried out. We conclude that due to the inherent flexibility of both melittin and CaM, their association can be characterized by an ensemble of conformers with various anchoring residues and different patterns of electrostatic interactions.

## Results and discussion

### Crystal structures of CaM–melittin complexes reveal three binding modes

**Figure 2:**
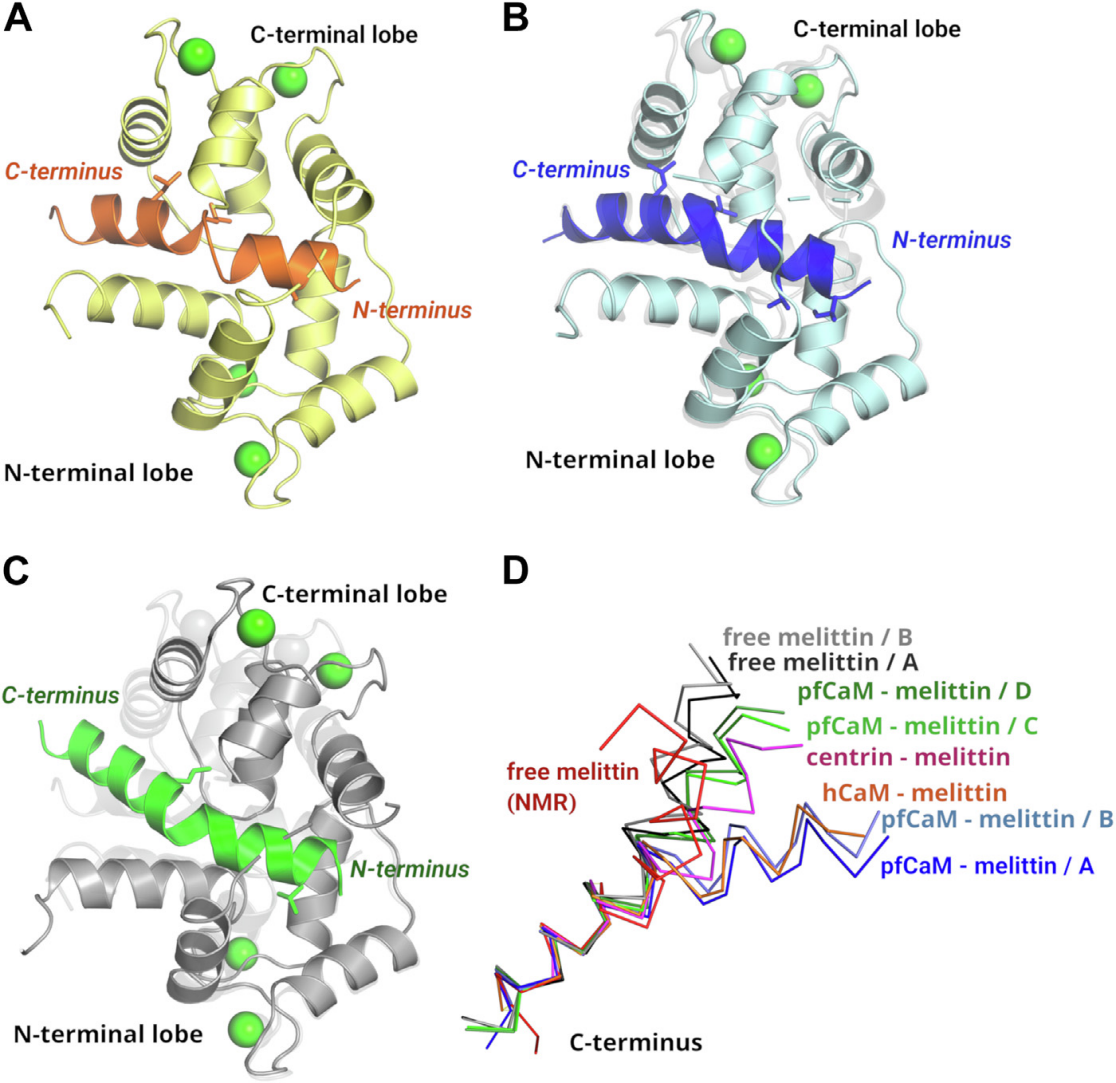
**Global conformation CaM**–**melittin complexes.***A*–*C*, hCaM–melittin (in *yellow* and *orange*, respectively) (*A*), pfCaM–melittin/A (in *light blue* and *dark blue*) (*B*), and pfCaM–melittin/C (in *dark gray* and *green*) (*C*) in the crystal structures are shown with the N-terminal lobes (*lower part* of the models) of CaM in the same orientation. In panels (*B*) and (*C*) structure of the hCaM–melittin complex is shown in transparent *gray* for reference, superimposed to the two pfCaM structures. Anchoring residues of melittin are shown as *sticks*. *D*, comparison of the conformation of melittin in its complexes with hCaM, pfCaM, *C. reinhardtii* centrin (PDB code: 3QRX) and in the crystal structure and NMR structure of the uncomplexed peptide (PDB codes: 2MLT and 6DST). CaM, calmodulin; hCaM, human calmodulin; pfCaM, Plasmodium falciparum calmodulin.

We solved the crystal structures of both hCaM and pfCaM complexed to melittin and refined them to 2.45 Å and 2.20 Å resolution, respectively. The stoichiometry of the complexes agrees with previously published results (31)—all complexes contain one CaM with one melittin bound in a parallel orientation, and four bound Ca^2+^ ions. The overall arrangement of the molecules in the complexes resembles the majority of CaM–peptide complexes, as CaM wraps around the helical peptide, forming a globular shape (Fig. 2, *A*–*C*).

The asymmetric unit of the hCaM–melittin complex contains a single entity while that of the pfCaM–melittin complexes contains four. The four pfCaM–melittin complexes (denoted as pfCaM complexes A, B, C, and D) belong to two, distinctly different binding modes of the same complex (Table S1): pfCaM/A (chains A/F and B/G belong to this group with a backbone RMSD of <1.0 Å) and pfCaM/C complex (chains C/H and D/I, with a similarly low RMSD). pfCaM/A and pfCaM/C complexes were chosen to represent the two binding modes and for the detailed structural analysis due to their better quality electron density maps in each pair (Table 1 and Fig. S1). While the structure of the hCaM–melittin complex shows more similarity with pfCaM/A complexes, it is still significantly different from either of the two pfCaM–melittin binding modes. Note, B-factors as well as the Wilson B-factors of the hCaM–melittin structure are unusually high considering the resolution of the dataset due to the quality of the crystals.

**Table 1:** Data collection and refinement statistics

Complex	pfCaM–melittin	Human CaM–melittin
Data collection		
Unit cell parameters *a*, *b*, *c*, (Å)	37.90, 60. 71, 68.47	40.53, 40.53, 349.14
*α*, *β*, *γ* (°)	95.75, 89.98, 97.05	90.00, 90.00, 120.00
Space group	P1	P6_1_22
Resolution (Å)	47.43–2.20 (2.30–2.20)	35.31–2.45 (2.55–2.45)
No. of unique refl./observed refl.	28,647/51,439	7039/70,910
<*I/σ(I)*>	8.78 (1.25)	19.46 (1.14)
*R*_meas_	0.090 (1.017)	0.051 (1.586)
Completeness (%)	93.5 (84.5)	98.4 (86.2)
CC(½)	99.8 (83.2)	99.8 (71.3)
Refinement		
Resolution range (Å)	47.43–2.20	35.31–2.45
*R*/*R*_free_ (No. of obs.)	0.234 (23814)/0.266 (1194)	0.267 (6342)/0.270 (310)
No. of non-hydrogen atoms: protein/Ca^2+^/solvent	4838/16/185	1146/4/3
B-factor of complexes A, B, C, D, solvent (Å^2^)	56.55, 53.57, 49.50, 70.95, 42.49	116.48, -, -, -, 91.64
Wilson B-factor (Å^2^)	44.96	98.91
RMS dev. bond length (Å)	0.003	0.003
RMS dev. bond angles (°)	0.543	0.723
Ramachandran fav./all./disall.	614/6/0	154/4/0

Data for the highest resolution shell are given in parentheses.

Due to the remarkable flexibility of its interdomain linker, CaM is known to be able to form complexes with highly different relative orientation of its N- and C-terminal lobes adapting to greatly varied target sequences (16). In the complexes determined herein, the relative orientation of the two lobes of CaM differs too, adjusting to the three different binding modes of melittin. The relative orientation of the two lobes can be characterized with the virtual dihedral angle defined by the four Ca^2+^ ions because the inner structure of the two Ca^2+^-binding lobes and thus the position of the 2 to 2 Ca^2+^ ions within them remain identical in different complexes (58). This dihedral angle is very similar in the pfCaM/A and hCaM complexes (118.0° and 117.5°, respectively), while it differs significantly for pfCaM/C, where it is 125.5°.

### Variable kink conformation of melittin in the complexes

While melittin adopts a predominantly α-helical structure in all complexes, its conformation is significantly different in CaM–melittin complexes with different binding modes (Fig. 2*D*). Analysis of the secondary structure of the peptide in the complexes with DSSP (59) showed that in the hCaM–melittin complex and in complex pfCaM/A two helical segments of melittin are intersected with a short turn in its so called kink region, while in melittin in the pfCaM/C binding mode even this segment remains part of the bent helical structure (Fig. 2*D* and Table S2).

The kink in the helical structure of melittin is caused by the presence of proline at position 14 (60) and was observed in all previously published structures of melittin (25). The bend angles (measured as the angle between the two helical segments between residues 3–11 and 15–22) observed in pfCaM–melittin and hCaM–melittin complexes are in the range of 140° to 158°. The angle value is larger in the hCaM and pfCaM/A complexes, similarly to the only known melittin complex structure (*C. reinhardtii* centrin–melittin complex), while the angle value in the pfCaM/C complex is smaller, more similar to those found in the uncomplexed forms of melittin (Table S3). Interestingly, the bending directions (relative to an ideal, linear helix) are also different; while melittin in binding modes pfCaM/A and hCaM bend in the same direction, it bends in a different direction in the pfCaM/C complex. The fact that both the domain orientation of CaM and the bending of melittin are similar in the hCaM and pfCaM/A complexes, but differ in the pfCaM/C complex, suggests that the sequence differences of hCaM and pfCaM might not have large effect on binding.

### Key elements of CaM–melittin interactions

Upon Ca^2+^-binding, hydrophobic residues of CaM become solvent accessible. These hydrophobic residues form a hydrophobic pocket in both lobes of CaM, which are usually occupied by bulky, hydrophobic anchor residues of the target peptide. Each CaM lobe contains a tetrad of hydrophobic residues, denoted FLMM residues (Phe19, Leu32, Met51, Met71 in the N-terminal hydrophobic pocket pfCaM contains Leu instead of Met at position 51 and Phe92, Leu105, Met124, Met144 in the C-terminal hydrophobic pocket), which were found to interact the anchoring residues of the target peptide in the majority of complexes (61).

**Figure 3:**
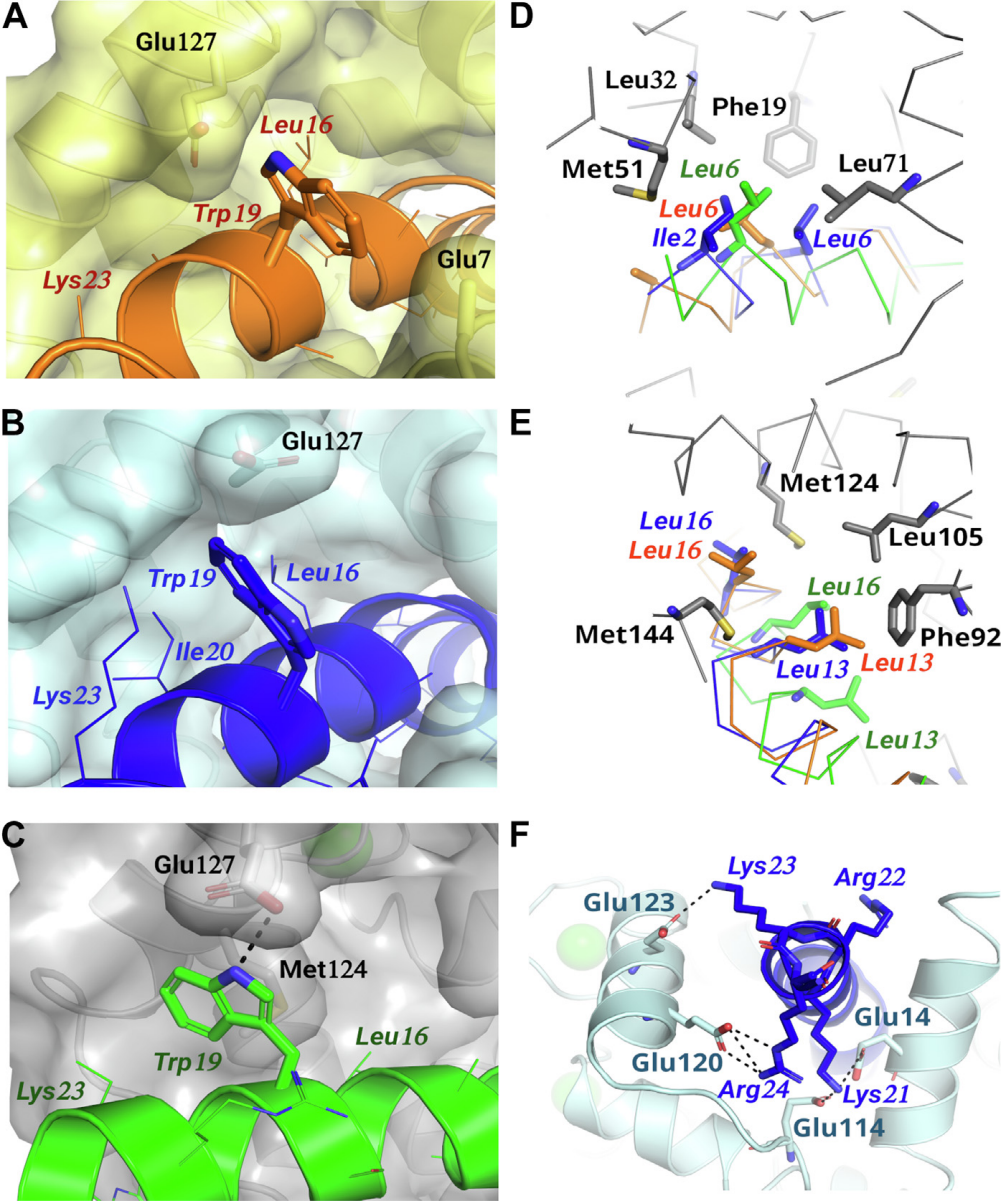
**CaM–melittin interactions in the crystal structures.***A*–*C*, the binding region of Trp19 side chain is outside of the hydrophobic pocket of CaM shown for (*A*) hCaM, (*B*) pfCaM/A, and (*C*) pfCaM/C, respectively. Residues of CaM that form contacts with Trp19 (<4.0 Å distance between nonhydrogen atoms) are shown as *sticks*. *D* and *E*, position of the potential N- (*D*) and C-terminal (*E*) anchoring residues of melittin relative to FLMM residues forming the hydrophobic pockets of CaM. Melittin molecules from complexes hCaM, pfCaM/A, and pfCaM/C are shown in *orange*, *blue*, and *green*, respectively. *F*, position of the charged residues of melittin and CaM near the C-terminal end of the binding channel in the pfCaM/A complex. Labels of melittin residues are shown as *italic*. CaM, calmodulin; hCaM, human calmodulin; pfCaM, *Plasmodium falciparum* calmodulin.

In the different CaM–melittin structures, different residues interact with the interior of the hydrophobic pockets (and therefore can be considered as anchoring residues) (Fig. 3 and Table S4). In the pfCaM/A complex, two residues form contacts with each hydrophobic pocket; Ile2 and Leu6 interact with the N-terminal hydrophobic pocket, while Leu13 and Leu16 forms hydrophobic contacts with the C-terminal hydrophobic pocket (Fig. 3, *D* and *E* and Table S4). In contrast to other CaM–peptide complexes, none of the mentioned residues are immersed deeply in either of the hydrophobic pockets. Based on the anchoring residues, this complex can be classified as a 1-5-12-15 type complex. In contrast, in the pfCaM/C complex, Leu6 and Leu16 could be identified as N-terminal and C-terminal anchoring residues, respectively. This conformation of the pfCaM–melittin complex can be classified as a 1-11 type CaM–peptide complex (Fig. 3, *D* and *E*).

Interestingly, while the overall conformation of the hCaM–melittin complex is more similar to the pfCaM/A complex, the interactions between hCaM and melittin represent a transition between the two pfCaM complexes. Leu6 is the sole anchoring residue in the N-terminal hydrophobic pocket, like in the pfCaM/C complex, while Leu13 and Leu16 contact the interior of the C-terminal hydrophobic pocket, as found also in the pfCaM/A complex. The hCaM–melittin complex therefore can be classified as a 1-8-11 type complex (Fig. 3, *D* and *E*).

Surprisingly, Trp19, the residue previously identified as the most likely C-terminal anchoring residue (32, 48, 62, 63), is partially accessible to the solvent in all complexes (Fig. 3, *A*–*C*). It contacts the exterior of the C-terminal hydrophobic pocket in pfCaM/C, while it is more solvent accessible for binding mode pfCaM/A and in the hCaM–melittin complex. The fact that the side chain of Trp19 is at least partially solvent exposed in all our crystal structures strongly suggests that it should be predominantly partially solvent exposed also in solution. The reported λ_max_ of 340 nm for the CaM–melittin complex (63) also indicates that Trp19 is only partially buried (64), which is consistent with our crystal structures.

The basic residues at the C terminus of melittin are located in the vicinity of several acidic side chains of CaM (Fig. 3*F*). In the pfCaM–melittin complexes, most of these side chains could be seen in electron density maps and form salt bridges with acidic side chains, while in the hCaM structure all basic side chains are disordered. Although melittin is in a different conformation and binding position in the different chains of the pfCaM complex, there are two salt bridges that could be observed in both conformations: Lys21 and Arg24 formed a salt bridge with Glu14, and Glu120, respectively in both pfCaM complexes.

Parallel orientation of melittin is its dominant orientation, though not exclusive; previous results indicated about 80%:20% of parallel: antiparallel ratio (47, 65). Our results suggest that the existence of a basic cluster containing four positively charged residues of melittin could be predominant in determining the binding orientation over the roles of possible anchoring residues. This hypothesis is also supported by the comparison of the hCaM–melittin complex with the CaM–TRPV1 (transient receptor potential cation channel subfamily V member 1) peptide complex ((22), PDB code: 3SUI), which latter shows CaM with almost identical backbone conformation but the peptide in the opposite—antiparallel—direction (Fig. S2). Basic residues are not clustered in the TRPV1 peptide, so possibly its orientation is not determined by electrostatic interactions, instead by the accommodation of the anchoring Trp residue for which the C-terminal pocket of CaM is the preferred binding site.

### Flexibility of the complexes explored by MD simulations

The fact that significantly different conformations of the same complex could be observed in crystals of the pfCaM–melittin complex, while only one conformation was seen in the hCaM–melittin complex prompted the question whether multiple binding modes might also exist in the hCaM–melittin system. The observation in the literature that chemical cross-linking derived distance restraints could not be fully satisfied by one model of the hCaM–melittin complex even when considering distance restraints specific to only one peptide orientation also suggested that flexibility and conformational dynamics may play a significant role in the interaction of CaM with melittin (33). Some of the residues participating in cross-linking found to be disordered in the crystal structures, making it difficult to compare the results. Specifically, most residues of the interdomain linker (residues 78–84) are missing from all models due to flexibility, and several other residues are also disordered in one or more structures. It is also worth noting that both Gly1 and the side chains of Lys21 and Lys23 are missing from the hCaM–melittin structure, making it impossible to validate any of the distance restraints. To elucidate the role of dynamics in the formation of CaM–melittin complexes, we performed molecular dynamics simulations starting from the crystal structure of hCaM–melittin and pfCaM–melittin A and C complexes. To gain better understanding of slow, large-scale motions of the systems, parallel simulations were performed for all complexes for a duration of 1500 to 4000 ns.

**Figure 4:**
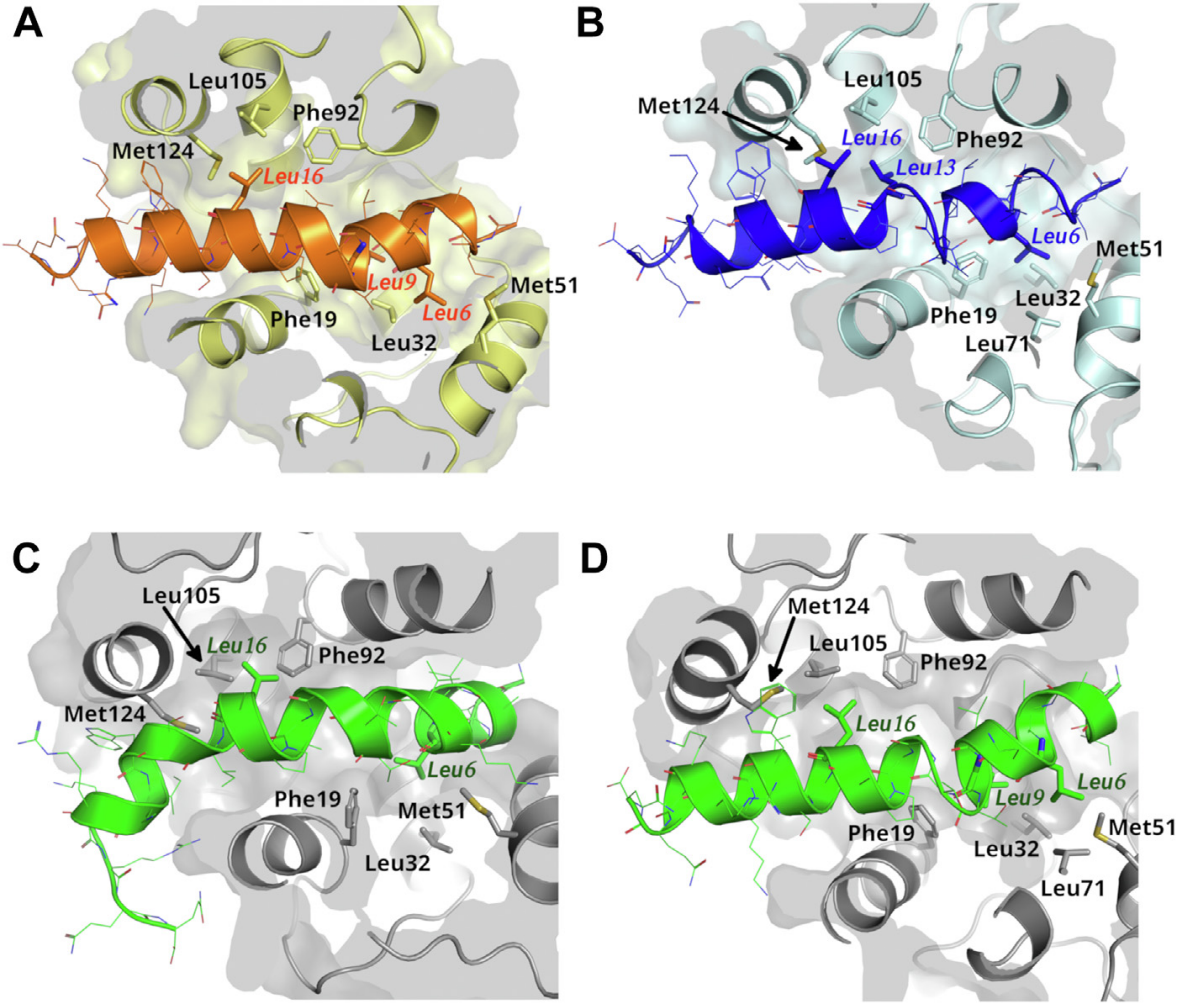
**Binding modes of melittin as explored by molecular dynamics simulations.***A*–*D*, mid-structures of the most populated clusters from the trajectories of simulations started from the crystal structure of the hCaM–melittin (*A*), pfCaM–melittin/A (*B*) and pfCaM–melittin/C (*C* and *D*, from the two parallel simulations) complexes. FLMM residues of CaM and anchoring residues of melittin are shown as *sticks*. Labels of melittin residues are *italic*. hCaM, human calmodulin; pfCaM, *Plasmodium falciparum* calmodulin.

The overall structure of the complexes remained stable throughout the simulations. The backbone RMSDs from the starting crystal structures stabilized in all trajectories at approximately 2.5 to 3.0 Å. Melittin and the four Ca^2+^ ions remained bound to CaM throughout the simulations, while CaM remained wrapped around melittin, which stayed in a predominantly helical conformation (Fig. 4).

To compare the simulated structures with their experimental counterparts, we calculated backbone RMSDs between all snapshots from the trajectories and the crystal structures of the three complexes (Figs. S3–S5) for the two components separately. When comparing structures of CaM, we obtained similar RMSD values with respect to all three experimental structures in most cases. Interestingly, in some trajectories, the lowest RMSDs could be obtained not with respect to the starting structure of the MD simulation (Figs. S4 and S5). This could be observed also when comparing experimental and simulated structures of melittin (Figs. S3–S5); the structures in most trajectories resembled melittin from the crystal structure of the hCaM–melittin complex most, even the ones that were started from other structures. The fact that the structure of both CaM and melittin was able to drift away from their respective starting structures and toward other crystal structures suggests that both complexes are similarly flexible and can be characterized by a similar conformational space.

Cluster analysis performed separately for CaM and melittin showed that the flexibility of both components remains even after complex formation (Fig. S6). The flexible linker of CaM allows the change of the relative orientation of its two domains (Fig. S6, *A*–*C*), which is necessary for the formation of different binding modes. Analysis of the CaM–melittin crystal structures also suggested that the flexibility of melittin is also essential for the changes of binding modes—cluster analysis of MD simulation confirmed this hypothesis, revealing that melittin also remained flexible during all simulations (Fig. S6*D*). Notably, conformers significantly different from either crystal structures also appeared within the most populated clusters, suggesting that the crystal structures may be snapshots from a large conformational space, rather than well-defined binding modes.

Multiple potential anchoring residues were found in the crystal structure of the pfCaM–melittin complex, while only one arrangement of anchoring side chains could be observed in hCaM–melittin crystals. To find out whether this occurred only due to differences in crystal packing (Table S5) or existing differences between the flexibility of the two complexes, we analyzed side chain interactions between melittin and the hydrophobic pockets of CaM in all trajectories (Table 2 and Figs. S7–S9). Interestingly, the arrangement of anchoring side chains deviated from those observed in the crystal structures which were used as starting points for the simulations. Specifically, the C-terminal anchoring residues remained the same as in the crystal structures, while the N-terminal anchoring residues changed in almost all cases (Table 2 and Fig. 4). In the hCaM–melittin complex, beside Leu6, Leu9 could be also observed to contact the interior of the N-terminal hydrophobic pocket during simulations. In the pfCaM/A complex, Leu6 emerged as the sole N-terminal anchoring residue—in contrast to the crystal structure, where Ile2 was also buried in the N-terminal hydrophobic pocket. Striking differences could be observed between the two parallel simulations started from the pfCaM–melittin/C structure; in one trajectory, Leu6 remained to be buried in the N-terminal hydrophobic pocket, while Leu9 was observed to be the N-terminal anchoring residue almost exclusively in the other trajectory.

**Table 2:** Variable anchoring residues of melittin

Complex ID	Crystal structure		MD simulation 1		MD simulation 2		MD simulation 3	
	N-lobe	C-lobe	N-lobe	C-lobe	N-lobe	C-lobe	N-lobe	C-lobe
hCaM	Leu6	Leu13, Leu16	Leu6, (Leu9)	Leu16, (Leu13)	Leu6, Leu9	Leu16, (Leu13)	Leu6, (Leu9)	Leu16, (Leu13)
pfCaM/A	Ile2, Leu6	Leu13, Leu16	Leu6	Leu13, (Leu16)	Leu6	Leu13, (Leu16)	n.a.	n.a.
pfCaM/C	Leu6	Leu16	Leu6	Leu16	Leu9, (Leu6)	Leu16	n.a.	n.a.

Anchoring residues of melittin in crystal structures and molecular dynamics simulations of hCaM– and pfCaM–melittin complexes. Evolution of the arrangements anchoring residues during the MD simulations are shown in Figs. S7–S9). Residues shown in parentheses were found to be in anchoring position in less than 10% of the frames of the trajectories.

A residue was considered as an anchoring residue if any of its side chain atoms were found to be closer than 2.5 Å to the center of mass of the side chains of the FLMM residues (and was verified by visual inspection).

While in some trajectories, both the N- and the C-terminal anchoring residues remained the same throughout the simulations, and in other cases, the arrangement of anchoring residues changed during the simulation (Figs. S7–S9). To study the structural background of swapping between anchoring residues, we analyzed the backbone clusters of the trajectories and also performed cluster analysis based on all nonhydrogen atoms in the N-terminal and C-terminal hydrophobic pockets (atoms that are closer than 5 Å to either of possible anchoring residues). We found that in some cases, both potential anchoring residues were in the vicinity of a pocket, therefore a minor rearrangement of side chains was enough to swap anchoring residues. In other cases, however, a shift in the position of melittin and a larger conformational change in the binding pockets resulted in changes of anchoring residues (see Fig. S10 for examples). Interestingly changes in CaM domain orientations or melittin bending angles did not always coincide with a change of anchoring residues.

The interactions formed by the basic cluster at the C terminus of melittin could not be fully studied in the crystal structures because some (or all in the case of hCaM, Table S4) of these side chains were disordered. This may suggest that similar to hydrophobic residues, which could swap the role of anchoring, some or all of these basic residues could participate in variable salt bridges instead of forming well-defined, specific interactions with CaM. Indeed, we found that almost all basic residues of melittin formed variable salt bridges/electrostatic interactions with CaM during the simulations. While some interactions were present in all trajectories of all three complexes, there were salt bridges that appeared only in some of the simulations and were completely absent from others (Table 3).

**Table 3:** Salt bridges formed by residues of the basic cluster in melittin

Complex ID	Melittin residue			
	Lys21	Arg22	Lys23	Arg24
hCaM	Glu11, **Glu14**, Glu114	Glu7, Glu11	**Glu123**	**Glu114**, **Glu120**
pfCaM/A	**Glu14**, **Glu114**	Glu7, Glu11	Glu123, Glu127	Glu120
pfCaM/C	**Glu11**, **Glu14**	Glu7, Glu11, Glu127	Glu120, Glu123, Glu127	**Glu14**, **Glu114**

Charged residue pairs with donor-acceptor distance less than 4 Å in at least 10% of all trajectories of a complex are listed (with bold: distance is less than 4 Å in at least 50% of the trajectories).

Tryptophan residues are the anchoring residues of the classical CaM target peptides described in early studies (66, 67) and in many complexes published so far. As such, the tryptophan (Trp19) residue of melittin was previously predicted to be the anchoring residue (68). Surprisingly, in the complexes determined here, Trp19 did not participate in anchoring, instead it was found to be partially exposed at the edge of the binding pocket of CaM (Fig. 3, *A*–*C*). During the molecular dynamics simulations, instead of participating in a rigid, well-defined set of interactions, it explored different conformations and interactions. A hydrogen bond between Trp19 and Glu127—also present in the pfCaM–melittin/C crystal structure—appeared in all simulations (in 7.8%, 44.1% and 36.2% in trajectories starting from the hCaM–melittin, pfCaM–melittin A and B complexes, respectively). A further possible hydrogen bond with Glu7 was present with low occurrence (17.4%) in simulations started from the hCaM–melittin structure. Further analysis of the crystal structures and the simulation trajectories revealed that accommodation of the rather hydrophobic tryptophan ring in a polar environment (surrounded by salt bridges) at the edge of the binding site is facilitated by shielding from either or both sides—by Met124, Glu127, or Ala128 of CaM and sidechain of Lys23 of melittin (Fig. 3, *A*–*C*). At least one of these shielding contacts (<4.0 Å distance between non-hydrogen atoms of side chains) appeared in more than 85% of the frames of all trajectories. The incomplete shielding of Trp19 might be why its orientation differs between the complexes in the crystal structures. The binding of a tryptophan residue outside the hydrophobic pocket, somewhat resembles CaM complexes formed with the small conductance calcium-activated potassium channel protein 2 (69, 70). There, the bound peptide segment contains a WXXXK motif ensuring partial shielding of the tryptophan from one side by the lysine, while it contacts Met124 or Ala88 at the other side (PDB structures 1G4Y, and 3SJQ. Note, in the case of 1G4Y the CaM-domain is in open conformation, similar to Ca^2+^-loaded domains, though it does not contain Ca^2+^ ions).

### Evolutionary differences of hCaM and pfCaM binding sites

**Figure 5:**
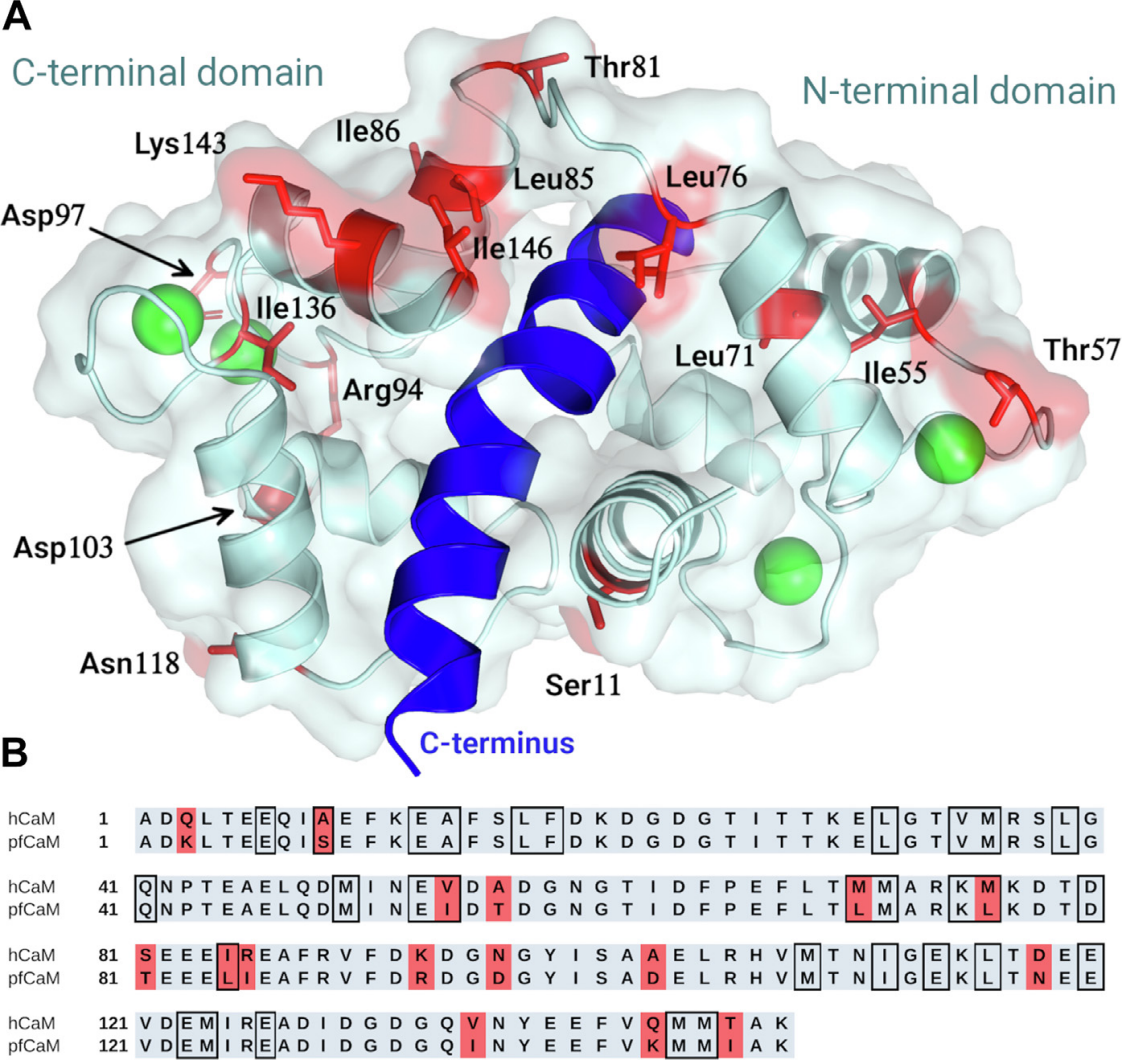
**Comparison of the sequence of pfCaM and hCaM.***A*, location of the differing residues (*red*) related to binding region of melittin (*blue*) is shown with the pfCaM–melittin/A complex. *B*, sequence alignment of pfCaM and hCaM. Residues contacting melittin (nonhydrogen atom distances closer than 4 Å) in either of the three binding modes found in the crystal structures are framed, differing residues are highlighted in *red*. hCaM, human calmodulin; pfCaM, *Plasmodium falciparum* calmodulin.

The primary structure of CaM is highly conserved, which is clearly illustrated by the fact that CaMs from two evolutionarily distant species, *P. falciparum* and *Homo sapiens*, show 89% sequence identity. Out of the 16 nonidentical residues, nine are similar substitutions, and only four participate in binding of melittin (Fig. 5). This highly similar primary structure may suggest that the structure and interaction properties of the two proteins should be very similar. Indeed, previously we found that the small molecule ligand binding properties of the two CaMs are practically identical (29). We also found however that clear differences could be observed in the thermodynamics of the peptide binding process of the two proteins; binding of melittin by pfCaM is enthalpically more preferred than by hCaM. The only differing residues that contribute to the intermolecular contacts between CaM and melittin are Met71 and Met76 of hCaM and corresponding Leu residues of pfCaM. Here, the shorter but branching and consequently more rigid residues of pfCaM might fit better to the shorter Thr and Leu side chains of melittin. Considering the multiple binding modes found in the crystal structures and ‘slippery’ nature of the anchoring during molecular dynamics simulations, we propose that these changes might be sufficient to explain the measured slight differences in the effectivity of binding.

Fromer and Shifman hypothesized that the balance of CaM specificity and promiscuity is fulfilled by different sets of residues within its binding site: they defined affinity-determining and specificity-determining residues using *in silico* methods (71). Interestingly, one of the residues differing between pfCaM and hCaM is affinity-determining, namely residue 71 (Met71 in hCaM and Leu71 in pfCaM), while none of the specificity-determining residues of pfCaM and hCaM differ. This is also in line with the observed minor difference in binding enthalpy and at the same time underlines that specificity and the well-defined nature of the binding mode are not necessarily correlated in the case of CaM.

### Revisiting low resolution models of CaM–melittin complexes

The structure of the hCaM–melittin complex was studied by different research groups using mass spectrometry combined with chemical cross-linking and limited proteolysis (21, 33, 47). This technique can provide distance restraints between cross-linked residues, which can aid low resolution model building. Interestingly, some of the published distance restraints were not satisfied in the crystal structures determined by us. However, in possession of the high resolution structures and the molecular dynamics trajectories, the distance restraints obtained by chemical cross-linking based methods (21, 33) could be used to refine our model describing the flexibility of the system. We note that while Scaloni *et al.* (21) found only cross-linked products characteristic to the parallel orientation of melittin, Schulz *et al.* (33) found cross-linked products originating from both orientations. Our crystal structures (and molecular dynamics trajectories) represent only the major orientation of the peptide; therefore, we considered only restraints that belong to the parallel arrangement.

**Figure 6:**
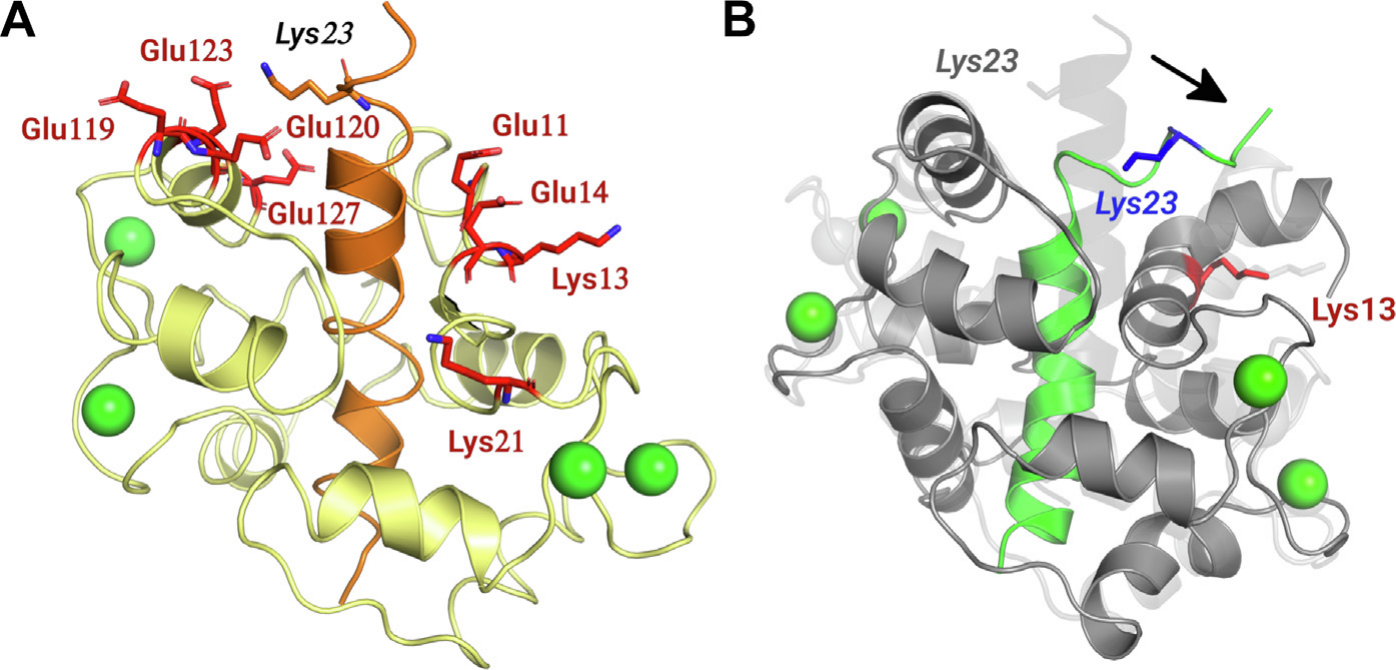
**Binding variants of the C-terminal part of melittin.***A*, location of Lys23 of melittin and residues of CaM which were reported to participate in possible cross-links with it, shown with the MD derived structure of the hCaM–melittin complex. *B*, conformational change required for the segment containing Lys23 of melittin to be within cross-linking distance to Lys13 of CaM. A frame from the MD simulation of the pfCaM/C binding mode is shown with *dark gray*/*green*, where the closest distance between Lys23 and Lys13 could be observed. The crystal structure of the pfCaM–melittin/C complex is displayed with transparent *light gray* for reference. Labels of melittin residues are in *italic*. CaM, calmodulin; pfCaM, *Plasmodium falciparum* calmodulin.

Most of the published residue–residue distances were found to be satisfied in a significant number of frames in the trajectories, but some residues came into sufficiently small distance from each other in only for a few short periods (Table 4). These distance restraints affected Lys23 from melittin, which is the closest residue to the C terminus of the peptide that can participate in cross-linking. While distance restraints of Lys23 which included CaM residues from its C-terminal lobe (Glu119, Glu120, Glu123, and Glu127) were readily satisfied in all trajectories, restraints including the N-terminal lobe of the protein (Glu11, Glu14, Lys23, and Lys21) were satisfied in only a few time frames of the simulations. These residues are located opposite to the cross-linking residues of the C-terminal lobe within the complex (Fig. 6*A*). All of these distance restraints therefore could not be satisfied at the same time by only one conformer and require a relatively large conformational change, a significant unfolding of the C-terminal part of melittin (Fig. 6*B*). The fact that there was a distance restraint that was satisfied in both parallel simulations of one complex and none of the parallel trajectories of the other complexes suggests that due to the possibility of large-scale conformational transitions a single MD simulation—even on the μs time scale—is not enough to adequately sample the conformational space available to CaM–melittin complexes. On the other hand, the similarity of the conformational transitions (changing of anchoring residues and salt bridge partners) observed during all the MD simulations and the highly conserved binding site residues suggest that the three experimentally determined CaM–melittin complex structures sample the similar conformational space of the hCaM–melittin and the pfCaM–melittin complexes.

**Table 4:** Testing multiple binding modes by MD simulations

Distance restraints derived from previous MS studies (21, 33) were checked through the molecular dynamics simulations. Restraints satisfied in a MD run are highlighted green, restraints close to being satisfied are highlighted with gray. In cases where multiple alternative CaM residues could be responsible for cross-linking (column 2), CaM residues found within the given distance to melittin residues are shown with twitches.

A restraint was considered satisfied if at least in one snapshot of MD the distance between the reactive groups of the side chains is smaller than the distance criteria associated with the given reagent as published (for reagents EDC, sulfo-DST and BS3, <5 Å, <8.5 Å and <13.4 Å, respectively).

Abbreviations: BS3, bis(sulfosuccinimidyl)suberate; EDC, [1-ethyl-3-(3-dimethylaminopropyl)carbodiimide hydrochloride; sulfo-DST, disulfosuccinimidyl tartarate.

^a^<8.7 Å.

^b^MS study of Schulz *et al.* (33) orientation A.

^c^MS study of Scaloni *et al.* (21).

## Conclusions

CaM is a widely studied example of transient hub proteins. Yet, target selection criteria of its Ca^2+^-dependent action is not fully understood. A consensus binding motif could not be determined and the inherent flexibility of interdomain organization and the shape adaptation potential of the hydrophobic pockets all contribute to its multispecificity. Melittin is widely used in CaM-related studies, often as a reference for CaM-binding and inhibition. While a number of studies provided low-resolution data on the binding interactions (33, 47), high-resolution structural information has not been available until now.

Our results presented here show that multiple binding modes can exist for CaM–melittin complexes, which seems to be an intrinsic characteristics of the association. This is probably made possible by multiple characteristics of melittin: (i) hydrophobic residues of similar size adjacent to each other in the melittin helix can act as alternative anchoring residues, (ii) its Trp residue is in a special configuration (WXXXK) which directs it to the C-terminal cluster of negative charges—releasing it from its anchoring position, (iii) the kink region of melittin separating its two anchors makes it more shape adaptable, and (iv) the C-terminal part of melittin can unfold within the complex and is possibly highly flexible in solution.

Although the binding modes shown here represent the dominant, parallel orientation of melittin, we suggest that flexible binding with alternative anchoring residues can also be sampled by minor binding modes. Our results indicate that anchoring residues are not so well defined, instead the electrostatic interactions could be the major determinant of melittin orientation—as the negatively charged clusters of CaM are more prominent on C-terminal end of the binding channel than on the N-terminal end, the parallel orientation becomes the dominant one.

Variations in binding mode of targets have already been detected in a few cases where typical examples (i) have a primary anchor residue fixed with the other anchoring site showing plasticity (19); (ii) swapping the electrostatic interactions of positively charged target residues between different sets of CaM residues (72); and (iii) the target peptide with palindromic segments of its sequence can bind in opposite orientations with the same anchoring residues (73). However, variability of target conformation, both anchor residues and sets of salt bridges for one particular target orientation, revealed in the melittin complexes herein is to our knowledge a unique feature.

In the case of melittin, it is important to note that besides its ability to bind to CaM, it has other functions too, so it can be considered as having a set of interacting residues not fully optimized for CaM binding but instead evolved for multiple functions. ITC studies (29, 65) showed dominant entropic contribution to the Gibbs energy of complex formation between CaM and melittin. The present model of variable and interchanging binding modes may partially explain this favorable entropic effect. Finding different complexes in the same crystal structure suggests that the nanomolar binding affinity is related to an ensemble of arrangements with similar stability—tight binding is achieved here not by optimized specific interactions but by simultaneously satisfying less optimal interaction patterns in co-existing different conformers.

## Experimental procedures

### Preparation of CaM–melittin complexes

pfCaM and hCaM were expressed in *E. coli* and purified using phenyl-sepharose affinity chromatography as described previously in detail (29). Both proteins were concentrated to 8 mg/ml (0.47 mM) in 5 mM CaCl_2_, 10 mM Hepes, pH 7.0 buffer before complex formation.

Lyophilized melittin was purchased from EZBiolab (Parsippany), and 10 mM solution of melittin was prepared by dissolving lyophilized melittin in 5 mM NaN_3_. CaM–melittin complexes were obtained by mixing CaM and melittin solutions in 1:1 molar ratio.

### Crystallization

Ca^2+^/pfCaM–melittin and Ca^2+^/hCaM–melittin complexes were crystallized by the hanging drop method at 20 °C.

Crystals of the Ca^2+^/pfCaM–melittin complex were obtained by mixing 2 μl protein solution (2.7 mg/ml protein complex of 1:1 molar ratio in 5 mM CaCl_2_, 10 mM Hepes buffer, pH 7.0) with 2 μl reservoir solution (0.25 M NaCl, 30 w/v% PEG3350, 0.1 M Bis-Tris buffer, pH 6.0). Thick, rod-shaped crystals grew within a week after setting up the plates. Crystals were quenched in liquid nitrogen without additional cryoprotectant.

Initial crystals of the hCaM–melittin complex were obtained by mixing 2 μl protein solution (2.7 mg/ml protein complex of 1:1 ratio in 5 mM CaCl_2_, 10 mM Hepes buffer, pH 7.0) with 2 μl reservoir solution (0.27 M NaCl, 27.5w/v% PEG3350, 0.1 M Bis-Tris buffer, pH 5.4). Small, hexagonal crystals appeared within a few days after setting up the drops. Crystal quality was improved by streak seeding into drops equilibrated over a reservoir solution containing 0.2 M NaCl, 22w/v% PEG3350, 0.1 M Bis-Tris, pH 5.4., using initial crystals as seed sources. Crystals were harvested into 0.2 M NaCl, 30w/v% PEG3350, 0.1 M Bis-Tris, pH 5.4, then quenched in liquid nitrogen.

### Data collection and structure determination

Diffraction data of the *P. falciparum* and hCaM–melittin complex crystals were collected at 100 K at beamlines PXIII at the Swiss Light Source (SLS) and ID23-1 at the European Synchrotron Radiation Facility (ESRF), respectively. Datasets were processed using the XDS package (74). Resolution limits were determined based on the *CC*_1/2_ values during data processing (75) and later validated by performing paired refinements. Use of weak data at the highest resolution shells resulted in unusually high *R*_meas_ but were justified by improvements of the quality of the electron density maps and improved models according to paired refinements.

Crystals of the Ca^2+^/hCaM–melittin complex were isostructural with the crystal structure of CaM in complex with a TRPV1 C-terminal peptide (PDB code: 3SUI). Initial electron density maps were calculated after refinement (rigid body, coordinates, individual atomic B-factor, torsion simulated annealing) using only CaM from 3SUI as initial model. The free R set from the dataset of 3SUI was used for refinement of the hCaM–melittin structure. After initial refinement, four Ca^2+^ ions and a large part of the alpha-helical melittin backbone appeared in the electron density maps, allowing manual model building. The electron density maps were of somewhat lower quality than expected at 2.45 Å resolution, which is explained by the unusually high Wilson B-factor and refined B-factors of the model (Table 1). Several other crystals of this complex grown from slightly different conditions were measured in order to find out if one with a higher degree of order could be found, but all datasets resulted in similar B-factors.

In the case of the pfCaM–melittin complex, phase problem was solved by molecular replacement using Molrep (76) and Phaser (77) of the CCP4 package (78). Separate searches were carried out using the N- and C-terminal domains of several CaM–peptide complexes. Best results were obtained by using separate CaM domains from the PDB entry 1CDL. After molecular replacement and initial rigid body refinement, bound Ca^2+^ ions and small parts of the bound alpha-helical peptide appeared in the electron density maps which allowed manual model building.

Manual model building was carried out with COOT (79), Phenix.refine (80), Refmac of the CCP4 package (81), and Buster (82) were used for refinement. Refinement included TLS refinement and refinement of coordinates and individual atomic B-factors. Direction of the alpha-helical peptides was determined based on the direction of the side chains. Sequence assignment of the peptide was based on the position of the loop region where the helix bends, the position of the two glycine residues and the position of bulky side chains. Waters were added to the model manually. The asymmetric unit of the pfCaM/melittin crystals contains four chains of Ca^2+^/pfCaM, all complexed with melittin (complexes (chains A/E, B/F, C/G and D/H of pfCaM/melittin), which represent two distinctly different binding modes. (Note, names of binding modes A, B, C and D throughout the text refer to the chain name of CaM in the crystal structure.) Therefore, torsion angle NCS restraints were applied pairwise for complexes A - B and C - D during refinement.

The N- and C-terminal residues and the interdomain linker region of CaM were disordered in both of the structures, therefore these residues were not included in the final models. Models were validated using the MolProbity server (83). Data collection and refinement statistics can be seen in Table 1. The structures of the hCaM–melittin and pfCaM–melittin complexes were deposited in the PDB with accession codes 8AHS and 8AHT respectively.

PyMOL was used for the calculation of interhelical angles and for creating the figures.

### Molecular dynamics simulations

All three binding modes were further analyzed by MD simulations using the Gromacs 5.1 simulation program (84) and the AMBER99SB-ILDNP force field (85). Missing residues and side chains were built into the models manually before the simulations. The complexes were embedded in dodecahedral boxes with 8 Å buffer distance. The boxes contained approximately 5700 TIP3P water molecules, as well as CaCl_2_ and NaCl in 0.05 mol/dm^3^ and 0.1 mol/dm^3^ concentrations, respectively. The net charge of the system was neutralized with sodium ions. Prior to simulations, energy minimization of the starting structures were performed with the L-BFGS method followed by the sequential relaxation of the restraints on the protein atoms in three, 200 ps long steps. An additional 200 ps long NVT simulation was performed in order to stabilize the pressure. Heavy atom–hydrogen bonds were constrained with the LINCS algorithm (84). Long-range electrostatic interactions were calculated with the Particle-Mesh Ewald method using a cutoff of 1.1 nm. Simulations were performed at 310 K by using the velocity rescale algorithm (86) and the pressure was kept at 1 bar using a Berendsen barostat. 2 to 3 parallel production runs were performed for all complexes for a duration of 1500 to 4000 ns using 2 fs time steps, during which snapshots were collected at every 4.0 ps. The beginning 200 to 300 ns of the simulations were discarded during data processing. Clustering analysis was performed with Gromacs using the gromos algorithm (87) based on the conformation of backbone atoms with 1.5 Å (for CaM and whole complex) or 1.0 Å (for melittin) RMSD cutoff.

## Data availability

The structures of the hCaM–melittin and pfCaM–melittin complexes were deposited in the PDB with accession codes 8AHS and 8AHT respectively. Any data not in the article can be shared upon request.

## Supporting information

This article contains supporting information.

## Conflict of interest

The authors declare that they have no conflicts of interest with the contents of this article.
