# Supplementary material for: Structures of calmodulin–melittin complexes show multiple binding modes lacking classical anchoring interactions
Source: J Biol Chem. 2023 Mar 9;299(4):104596. doi: 10.1016/j.jbc.2023.104596 (PMC10140167; doi:10.1016/j.jbc.2023.104596)
Supplement: Supporting Figures S1–S10 and Tables S1–S5 [file mmc1.pdf]

Supporting information for

Structures of calmodulin–melittin complexes show multiple binding modes lacking classical anchoring interactions

Zsolt Dürvanger[1], Tünde Juhász[2], Károly Liliom[3], Veronika Harmat[1,4]*

[1] Laboratory of Structural Chemistry and Biology, Institute of Chemistry, ELTE Eötvös Loránd University, Budapest, Hungary
[2] Institute of Materials and Environmental Chemistry, Research Centre for Natural Sciences, Budapest, Hungary
[3] Department of Biophysics and Radiation Biology, Faculty of Medicine, Semmelweis University, Budapest, Hungary
[4] ELKH-ELTE Protein Modelling Research Group, Eötvös Loránd Research Network, Budapest, Hungary

Corresponding author: Veronika Harmat
*e-mail: veronika.harmat@ttk.elte.hu

This file includes:

Figures S1 – S10
Tables S1 – S5

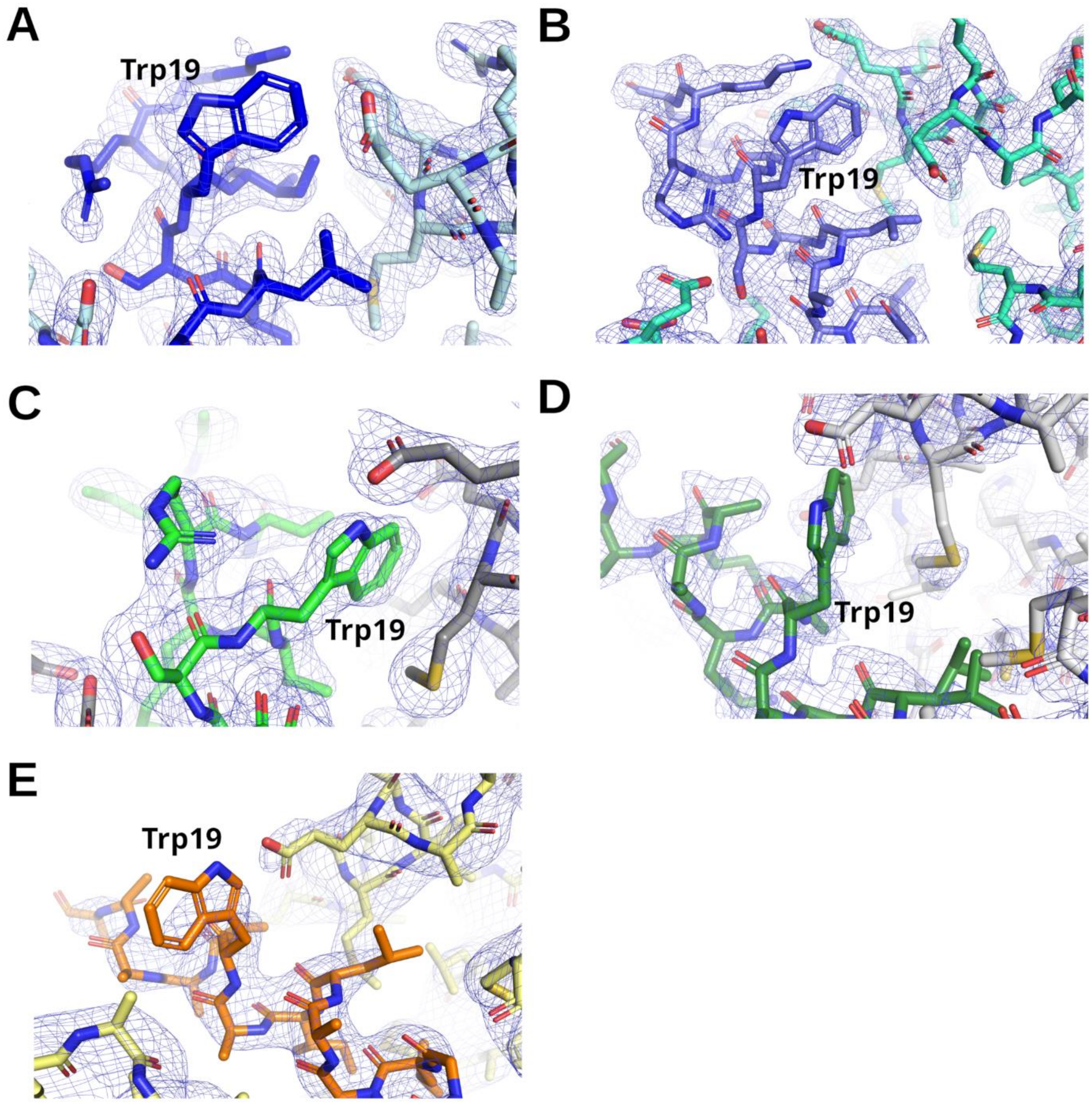

Supporting figure S1. Representative sections of the $2mF_o$-DF_c electron density maps of pfCaM–melittin complexes A, B, C, D (**A-D**) and the hCaM–melittin complex (**E**) contoured at 1.0 σ level.

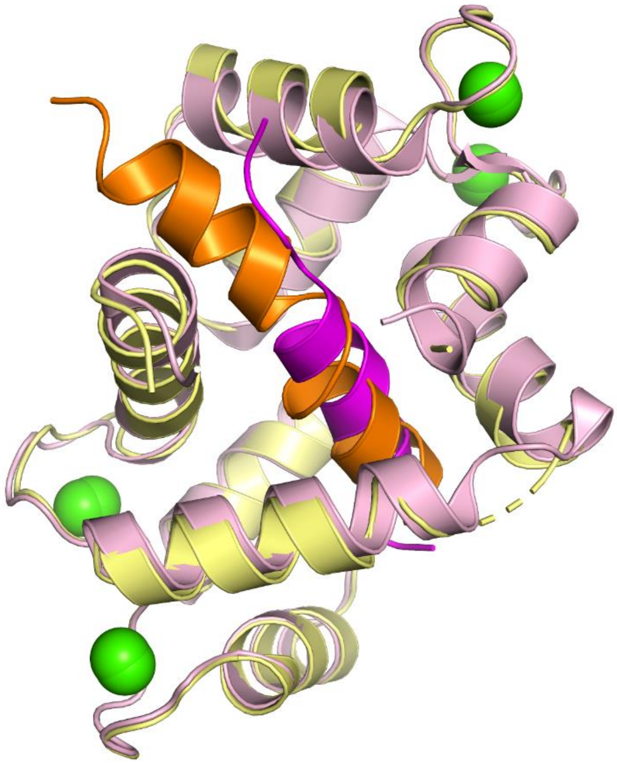

Supporting figure S2. Comparison of the conformation of the final model of the hCaM–melittin complex (CaM: yellow, melittin: orange, Ca^{2+} ions: green) with the complex used as search model during molecular replacement (CaM: light pink, TRPV1 C-terminal peptide: magenta, PDB code: 3SUI)

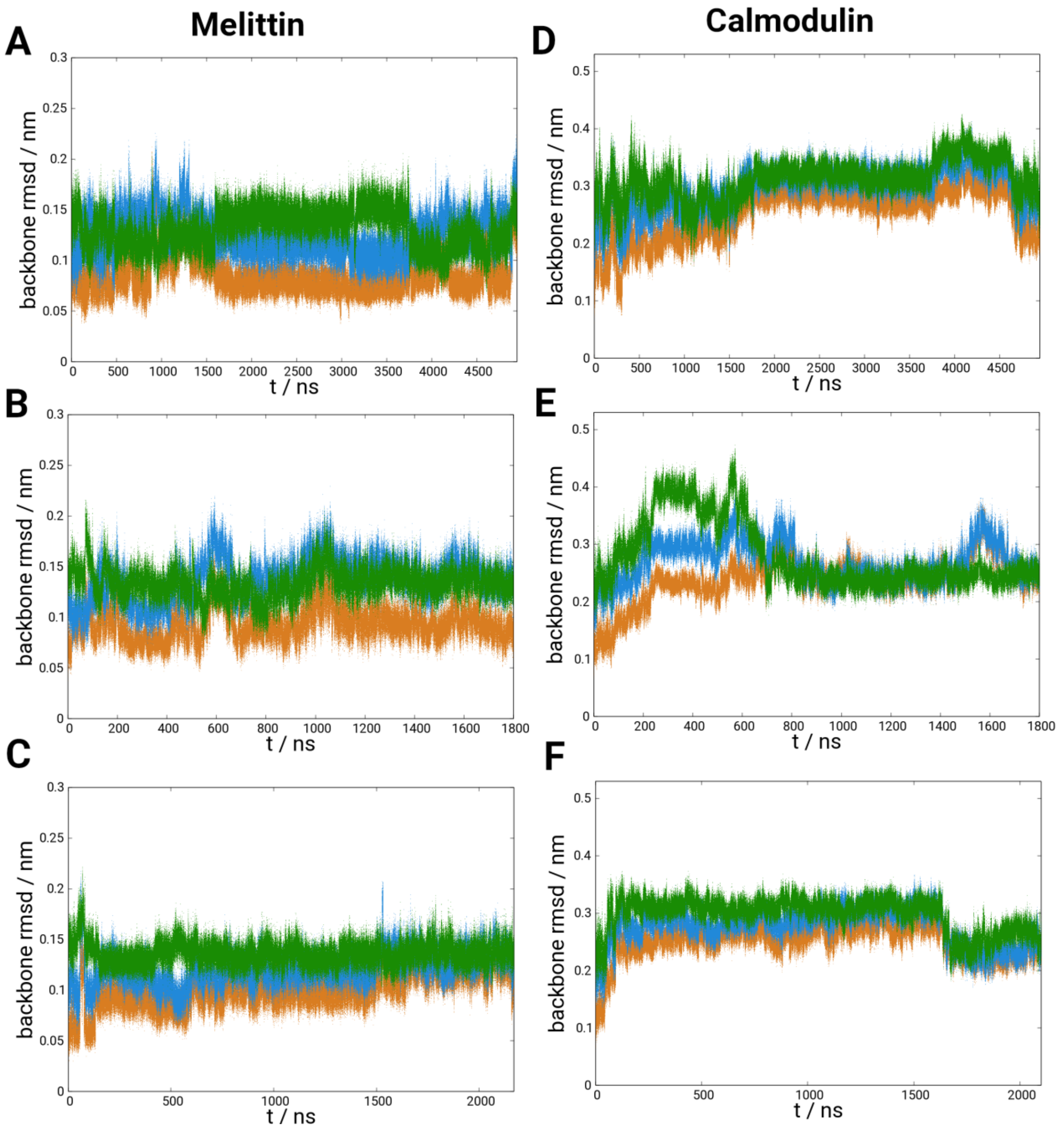

Supporting figure S3. Backbone RMSD between structures from the three trajectories started from the hCaM – melittin structure and the crystal structures (orange: hCaM – melittin, blue: pfCaM – melittin / A, green: pfCaM – melittin / C) calculated for backbone atoms of melittin (**A, B, C**) and CaM (**D, E, F**) separately. In order to elucidate the role of the flexibility of CaM and melittin in complex formation, we computed RMSDs to the two components separately, presented in Supporting figures S3-S5. To obtain comparable values, RMSDs were calculated only for residues that could be modelled in all crystal structures (CaM residues 4-73 and 86-145, melittin residues 3-24).

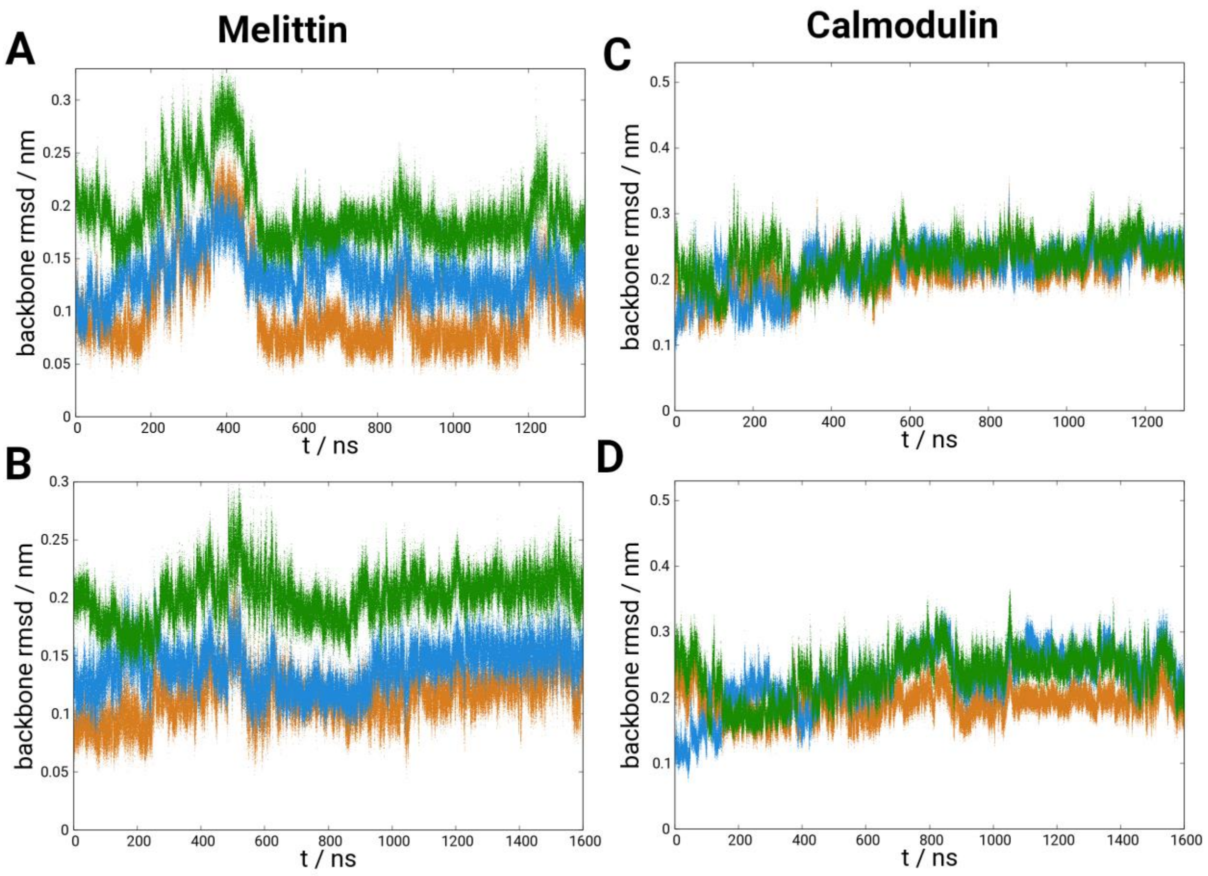

Supporting figure S4. Backbone RMSD between structures from the two trajectories started from the pfCaM – melittin / A structure and the crystal structures (orange: hCaM – melittin, blue: pfCaM – melittin / A, green: pfCaM – melittin / C) calculated for backbone atoms of melittin (**A, B**) and CaM (**C, D**) separately. Note, that based on the RMSD values in the second MD simulation, the complex resembles more to the hCaM – melittin crystal structure, than to its starting structure.

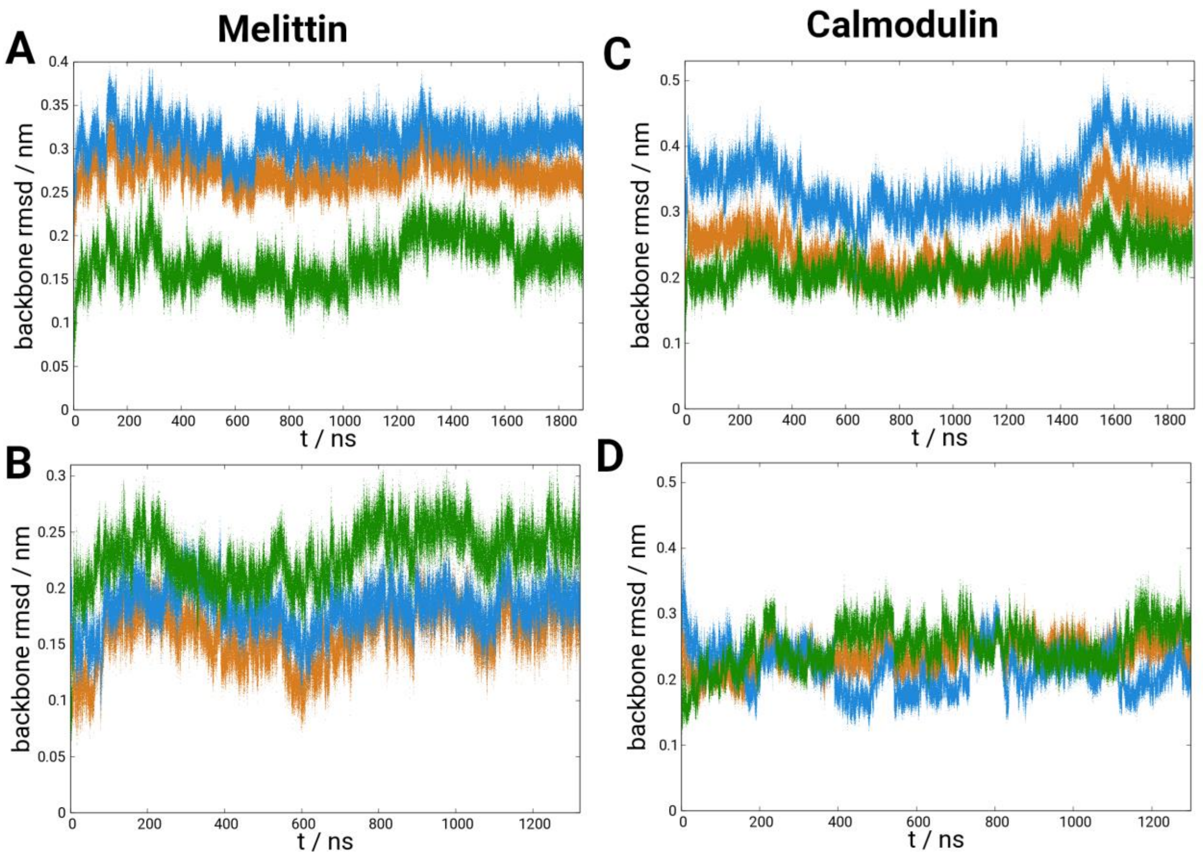

Supporting figure S5. Backbone RMSD between structures from the three trajectories started from the pfCaM – melittin / C structure and the crystal structures (orange: hCaM – melittin, blue: pfCaM – melittin / A, green: pfCaM – melittin / C) calculated for backbone atoms of melittin (**A, B**) and CaM (**C, D**) separately. It is interesting to compare the two trajectories started from the pfCaM – melittin / C structure: in the first trajectory, melittin and CaM both remained most similar to the pfCaM – melittin / C crystal structure. In the second trajectory on the other hand, similar RMSDs were obtained by using either crystal structures as reference, while melittin resembled the other two crystal structures more, than its starting structure.

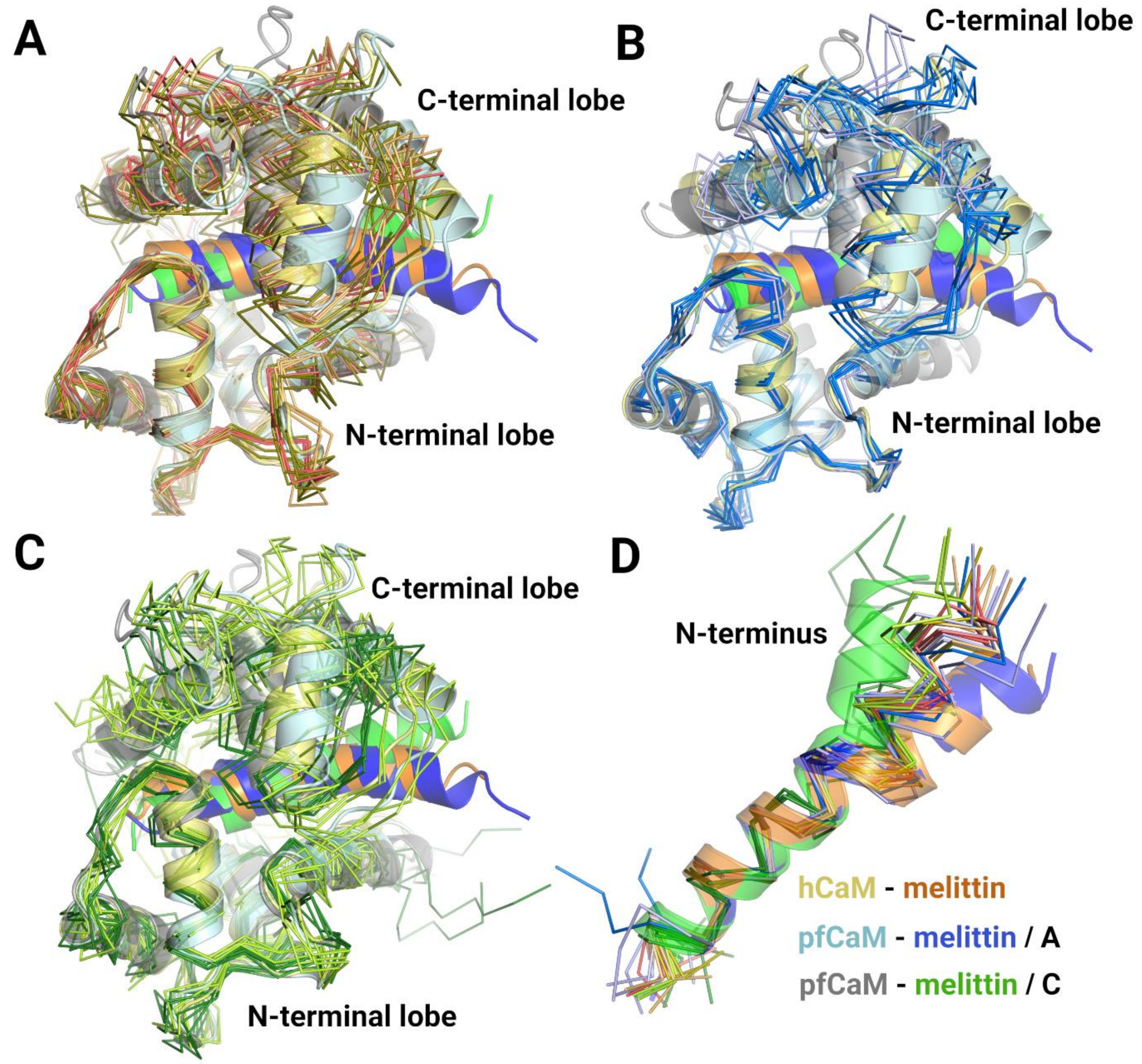

Supporting figure S6. CaM and melittin flexibility during MD simulations. Comparison of main backbone clusters (representing at least 5% of the snapshots) of CaM in trajectories started from the hCaM – melittin (**A**), pfCaM – melittin / A (**B**) and pfCaM – melittin / C (**C**) crystal structures. Crystal structures are presented in cartoon representation for reference (hCaM – melittin: yellow and orange, pfCaM – melittin / A – light and dark blue, pfCaM – melittin / B – gray and green). Mid structures of clusters from different trajectories are shown as ribbons and colored in different shades of the same color. The N-terminal domains of the CaM structures are superimposed. **D**. Comparison of main backbone clusters of melittin with the three crystal structures. Models are colored similarly to figures **A-C**.

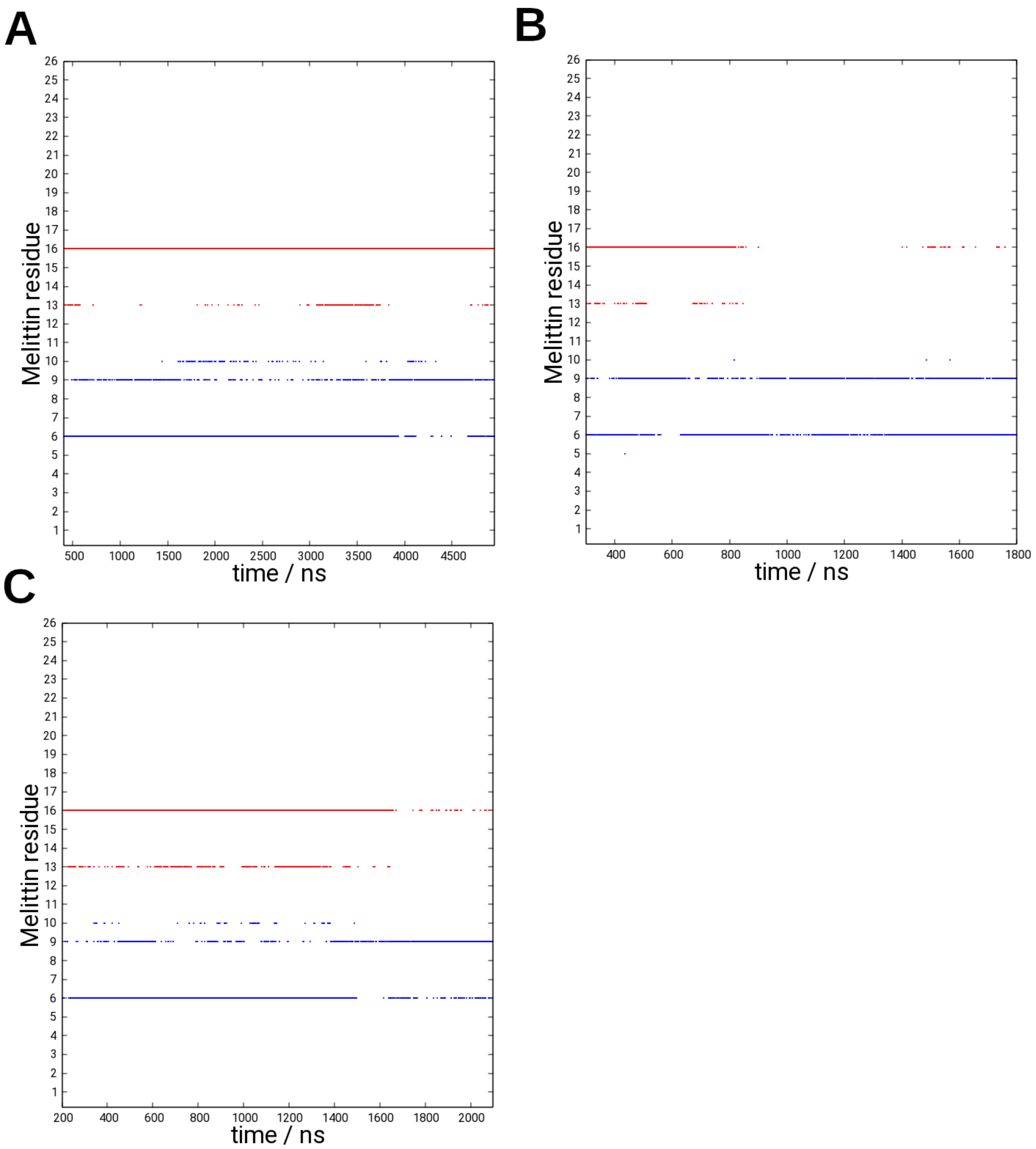

Supporting figure S7. Evolution of the arrangement of anchoring residues during the 3 parallel MD simulations started from the crystal structure of the hCaM–melittin complex. Melittin residues anchoring in the pockets of N- and C-terminal domains CaM are shown with blue and red, respectively.

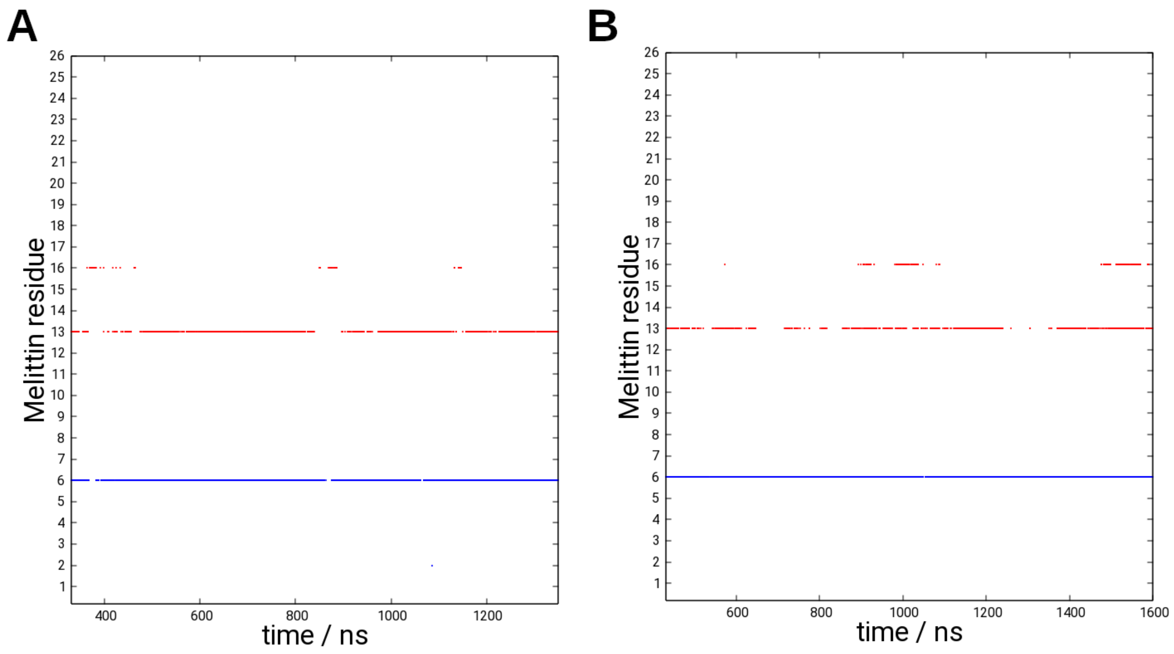

Supporting figure S8. Evolution of the arrangement of anchoring residues during the 2 parallel MD simulations started from the crystal structure of the pfCaM–melittin/A complex. Melittin residues anchoring in the pockets of N- and C-terminal domains CaM are shown with blue and red, respectively.

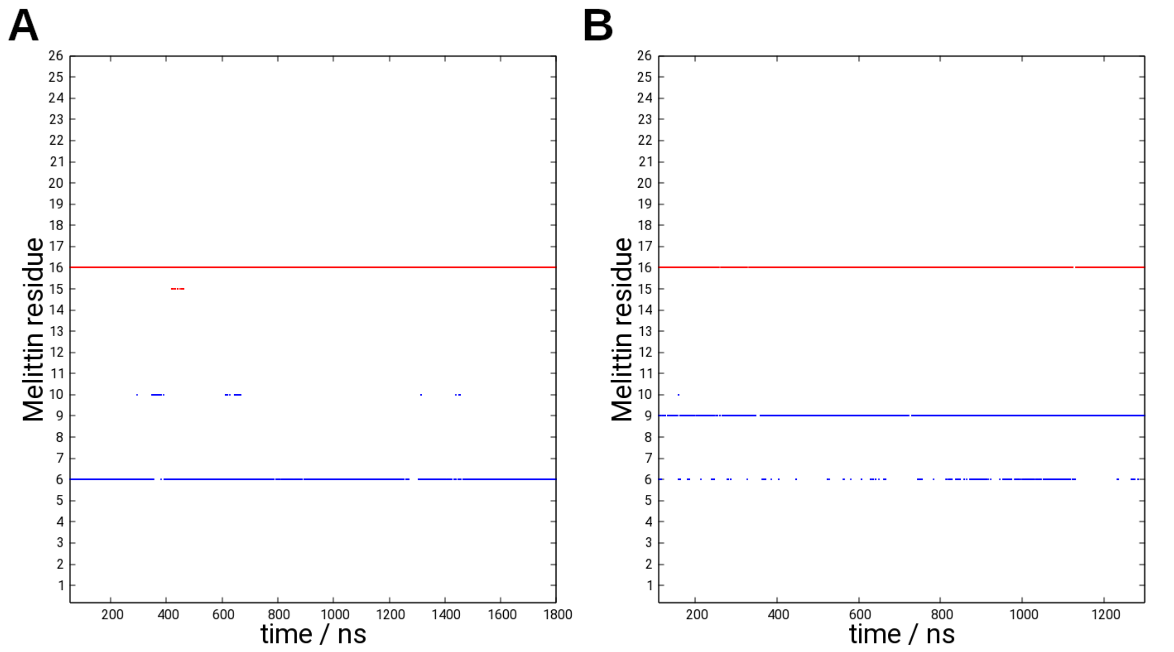

Supporting figure S9. Evolution of the arrangement of anchoring residues during the 2 parallel MD simulations started from the crystal structure of the pfCaM– melittin/C complex. Melittin residues anchoring in the pockets of N- and C-terminal domains CaM are shown with blue and red, respectively.

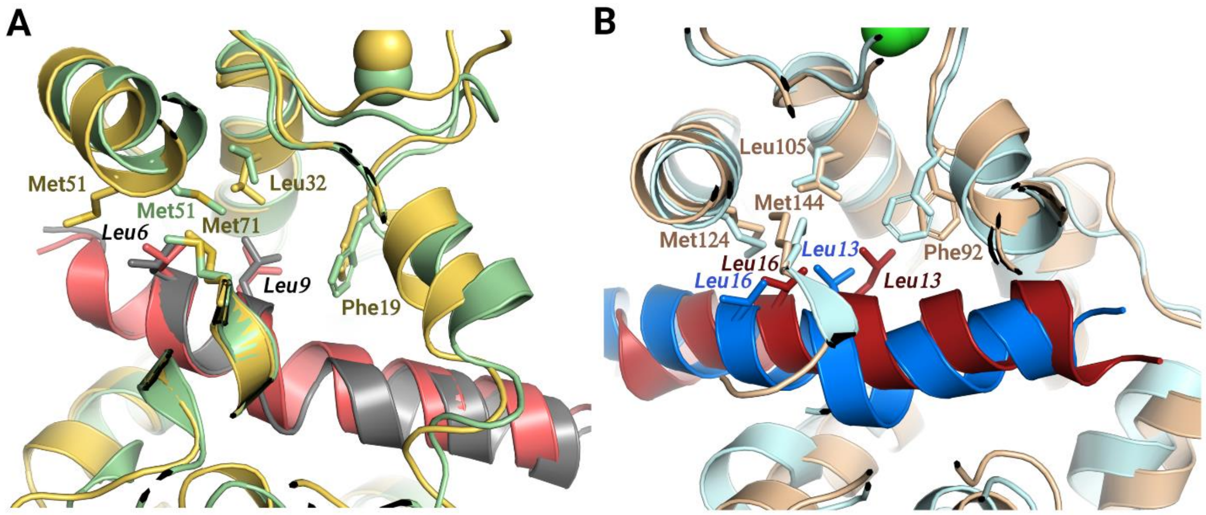

Supporting figure S10. **A**. Comparison of the first (yellow / red) and second (green / gray) most populated N-terminal binding pocket clusters of the first trajectory started from the hCaM – melittin crystal structure. Rearrangement of hydrophobic residues forming the pocket allowed Leu9 to participate in anchoring. **B**. Comparison of the first (light blue / dark blue) and second (salmon / red) most populated C-terminal binding pocket clusters of the first trajectory started from the pfCaM – melittin / A crystal structure. Shifting of the helical melittin with respect to the C-terminal lobe of CaM and a minor rearrangement of side chains of the binding pocket allowed Leu16 to participate beside (and in some snapshots instead) of Leu13.

Supporting table S1. Backbone RMSD between CaM–melittin complexes from our crystal structures. RMSD was calculated for backbone atoms of residues that are present in all 5 models. (Color code: RMSD< 1.0 Å: green, conformation can be considered as practically identical; 1.5 Å<RMSD<2.0 Å: pink, similar conformation with notable differences in molecular contacts; RMSD<2.0 Å: red, significant difference both in CaM domain orientation and in molecular contacts.

	**hCaM–melittin**	**pfCaM–melittin/A**	**pfCaM–melittin/B**	**pfCaM–melittin/C**	**pfCaM–melittin/D**
**hCaM–melittin**		1.540 Å	1.776 Å	2.367 Å	2.506 Å
**pfCaM–melittin/A**	1.540 Å		0.557 Å	2.564 Å	2.597 Å
**pfCaM–melittin/B**	1.776 Å	0.557 Å		2.714	2.701 Å
**pfCaM–melittin/C**	2.367 Å	2.564 Å	2.714 Å		0.765 Å
**pfCaM–melittin/D**	2.506 Å	2.597 Å	2.701 Å	0.765 Å	

Supporting table S2. Secondary structure of melittin crystal structures of hCaM and pfCaM–melittin complexes calculated by DSSP. α-helical regions are shown with blue, hydrogen bonded turn regions are shown with orange and bend regions are shown with green colors. Irregular regions are left with white backgrounds. Residues missing from the models are colored with black.

**Melittin residue**		**CaM–melittin complexes from crystal structures**				
No.	type	hCaM–melittin	pfCaM–melittin/A	pfCaM–melittin/B	pfCaM–melittin/C	pfCaM–melittin/D
1	Gly	-	irregular	irregular	-	-
2	Ile	irregular	helical	helical	-	-
3	Gly	helical	helical	helical	irregular	irregular
4	Ala	helical	helical	helical	helical	helical
5	Val	helical	helical	helical	helical	helical
6	Leu	helical	helical	helical	helical	helical
7	Lys	helical	helical	helical	helical	helical
8	Val	helical	helical	helical	helical	helical
9	Leu	helical	helical	helical	helical	helical
10	Thr	helical	bend	turn	helical	helical
11	Thr	helical	bend	turn	helical	helical
12	Gly	turn	helical	helical	helical	helical
13	Leu	helical	helical	helical	helical	helical
14	Pro	helical	helical	helical	helical	helical
15	Ala	helical	helical	helical	helical	helical
16	Leu	helical	helical	helical	helical	helical
17	Ile	helical	helical	helical	helical	helical
18	Ser	helical	helical	helical	helical	helical
19	Trp	helical	helical	helical	helical	helical
20	Ile	helical	helical	helical	helical	helical
21	Lys	helical	helical	helical	helical	helical
22	Arg	turn	turn	helical	helical	helical
23	Lys	irregular	turn	turn	helical	helical
24	Arg	irregular	irregular	irregular	helical	helical
25	Gln		irregular		helical	helical
26	Gln				irregular	irregular

Supporting table S3. Interhelical angles in CaM–melittin crystal structures and published melittin crystal structures. Angles between helical segments defined by melittin residues 3-11 and 15-22 were calculated using PyMol.

**Structure**	**Interhelical angle**
hCaM–melittin	155.29°
pfCaM–melittin/A	157.90°
pfCaM–melittin/B	153.45°
pfCaM–melittin/C	144.09°
pfCaM–melittin/D	139.55°
Chlamydomonas reinhardtii centrin - melittin	155.70°
free melittin (crystal structure) / A	131.52°
free melittin (crystal structure) / B	128.30°
free melittin (NMR structure, average)	134.51°

Supporting table S4. Main types of interactions formed between CaM and melittin in the crystal structures. For the anchoring residues, contacting residues are listed with number of contacts of the anchoring side chain (<4.0 Å distance of non-hydrogen atoms). For the cluster of positively charged residues hydrogen bonds are listed (<3.5 Å D..A distance). FLMM residues are shown in bold.

**Complex**	**Residues of melittin**	**Contacting residues of CaM**	**Nr. of contacts /H-bonds**
pfCaM/A	*N-terminal anchoring residues:*		
	Ile2	**Leu32**, Met36, **Met51**, Lys75	5
	Leu6	**Leu71**, Lys75, Leu76	3
	C-terminal anchoring residues:		
	Leu13	Met109, **Met124**, Met145	4
	Leu16	**Met124**, Glu127	6
	Charged residues		6
	Lys21	Glu14, Glu114	
	Arg22	-	
	Lys23	Glu123	
	Arg24	Glu120, Lys115 (carbonyl oxygen)	
pfCaM/C	*N-terminal anchoring residue:*		
	Leu6	**Phe19**, Met36, Ile55	4
	C-terminal anchoring residue:		
	Leu16	Met109, **Met124**	3
	Charged residues		5
	Lys21	Glu14	
	Arg22	-	
	Lys23	-*	
	Arg24	Glu120, Lys115 (carbonyl oxygen)	
Human CaM	*N-terminal anchoring residue:*		
	Leu6	-*	
	C-terminal anchoring residues:		
	Leu13	Met109, **Met124**	4
	Leu16	Ala128, **Met144**	2
	Charged residues:		
	Lys21, Arg22, Lys23, Arg24	-*	

*Side chain of melittin residue is disordered or partially disordered

Supporting Table S5. Number of atoms involved in crystal contacts between neighboring complexes. After removing H atoms, atoms of the complex within 4.0Å distance of neighboring complexes within the crystal were counted. *

	Number of atoms contacting neighboring complexes			List of melittin residues contacting neighboring complexes**
complex	CaM N-terminal domain	CaM C-terminal domain	melittin	
hCaM - melittin	42	52	0	-
pfCaM- melittin /A	79	50	6	Arg24, **Gln25**
pfCaM- melittin /B	79	49	14	Trp19, Arg22, Lys23***
pfCaM- melittin /C	110	53	9	Lys21, Arg24, **Gln25**, **Gln26**
pfCaM- melittin /D	109	51	16	Gly3, Lys21, Arg24, **Gln25**, **Gln26**

* Crystal contacts might influence the structure of CaM – melittin complex in two ways: i) As most part of melittin is buried by CaM, melittin residues only at the ends of the peptide reach outside the complex, either stabilized by crystal contacts or being disordered, so the helical conformation of the end of these end chains could be crystallographic artefacts (Gln25, Gln26). ii) Different shapes of the complex (i.e. different orientations of CaM domains) within the crystal suggest that multiple binding conformations of similar stability may co-exist in solution – in that case the crystal contacts of both CaM domains may select, which of those alternative complex shapes are stabilized in the crystal lattice.

** Shown underlined: melittin residues having multiple contacts with CaM within their complex, in contrast having few contacts outside their complex; in bold: melittin residues contacting only the neighboring complexes.

***Last ordered residue of melittin (Arg24) shows multiple contacts with CaM within the complex.
